# Anti–PD-1 chimeric antigen receptor T cells efficiently target SIV-infected CD4^+^ T cells in germinal centers

**DOI:** 10.1172/JCI169309

**Published:** 2024-04-01

**Authors:** Karsten Eichholz, Yoshinori Fukazawa, Christopher W. Peterson, Francoise Haeseleer, Manuel Medina, Shelby Hoffmeister, Derick M. Duell, Benjamin D. Varco-Merth, Sandra Dross, Haesun Park, Caralyn S. Labriola, Michael K. Axthelm, Robert D. Murnane, Jeremy V. Smedley, Lei Jin, Jiaxin Gong, Blake J. Rust, Deborah H. Fuller, Hans-Peter Kiem, Louis J. Picker, Afam A. Okoye, Lawrence Corey

**Affiliations:** 1 Vaccine and Infectious Disease Division, Fred Hutchinson Cancer Center, Seattle, Washington, USA.; 2 Vaccine and Gene Therapy Institute and Oregon National Primate Research Center (ONPRC), Oregon Health & Science University, Beaverton, Oregon, USA.; 3 Stem Cell and Gene Therapy Program, Fred Hutchinson Cancer Center, Seattle, Washington, USA.; 4 Department of Laboratory Medicine and; 5 Department of Medicine, University of Washington, Seattle, Washington, USA.; 6 Washington National Primate Research Center (WaNPRC), Seattle, Washington, USA.; 7 Department of Microbiology, University of Washington, Seattle, Washington, USA.

**Keywords:** AIDS/HIV, Therapeutics, Gene therapy, Immunotherapy, T cells

## Abstract

Programmed cell death protein 1 (PD-1) is an immune checkpoint marker commonly expressed on memory T cells and enriched in latently HIV-infected CD4^+^ T cells. We engineered an anti–PD-1 chimeric antigen receptor (CAR) to assess the impact of PD-1 depletion on viral reservoirs and rebound dynamics in SIVmac239–infected rhesus macaques (RMs). Adoptive transfer of anti–PD-1 CAR T cells was done in 2 SIV-naive and 4 SIV-infected RMs on antiretroviral therapy (ART). In 3 of 6 RMs, anti–PD-1 CAR T cells expanded and persisted for up to 100 days concomitant with the depletion of PD-1^+^ memory T cells in blood and tissues, including lymph node CD4^+^ follicular helper T (TFH) cells. Loss of TFH cells was associated with depletion of detectable SIV RNA from the germinal center (GC). However, following CAR T infusion and ART interruption, there was a marked increase in SIV replication in extrafollicular portions of lymph nodes, a 2-log higher plasma viremia relative to controls, and accelerated disease progression associated with the depletion of CD8^+^ memory T cells. These data indicate anti–PD-1 CAR T cells depleted PD-1^+^ T cells, including GC TFH cells, and eradicated SIV from this immunological sanctuary.

## Abstract 2

Anti-PD-1 chimeric antigen receptor T cells expand and efficiently target Simian immunodeficiency virus-infected CD4+ T cells in germinal centers in rhesus macaques.

## Introduction

HIV infects cells of the immune system through interaction with CD4, CCR5, and CXCR4 and establishes long-lasting infections that develop into the acquired immunodeficiency syndrome if left untreated. With the advent of antiretroviral therapy (ART), HIV infection has turned into a life-long controllable disease. However, residual viral transcription and translation, immune activation, and inflammation are associated with increased risk for comorbidities, such as blood and solid cancers, HIV-associated neurological disorders, and cardiovascular diseases, in people living with HIV. This highlights the need to develop strategies for curing HIV infection, and recent modeling data suggest curative interventions could contribute to controlling the HIV epidemic (1, 2). During ART, HIV-infected cells mainly reside in lymphoid tissues, including immune-privileged sites, such as lymph node (LN) B cell follicles and gut-associated lymphoid tissues (3–8). Importantly, PD-1^+^ CD4^+^ T cells and, more specifically, follicular helper T (TFH) cells, which are characterized by high programmed cell death protein 1 (PD-1) and CXCR5 expression, are major sites for HIV/SIV infection and persistence during ART. In LNs, rectal tissue, and peripheral blood of ART-suppressed individuals, PD-1^+^ CD4 T cells harbor the majority of replication- and translation-competent inducible provirus (9–13). Moreover, PD-1–expressing central, transitional, and effector memory CD4^+^ T cells produce more viral particles upon reactivation than their PD-1 counterparts (11), and ex vivo and in vitro data indicate that HIV preferentially replicates in TFH cells (14). Similarly, circulating peripheral TFH cells show the highest HIV gag RNA and protein expression levels in viremic individuals ex vivo (15). Taken together, these data suggest that targeted depletion of PD-1^+^ CD4^+^ T cells may be an effective strategy for limiting HIV persistence.

One approach to targeting and eliminating PD-1^+^ T cells in vivo is the use of chimeric antigen receptor (CAR) T cells. CAR T cells include chimeric proteins engineered to redirect the killing function of T cells toward cell-surface proteins independently of MHC-I and -II presentation. Structurally, these are single-pass transmembrane receptors that consist of a single-chain variable fragment (scFv) ectodomain for target recognition, one single transmembrane domain, and in most cases, 2 intracellular domains that mediate T cell activation and cytolytic functions through a CD3ζ chain downstream of a CD28- or 4-1BB–derived costimulatory domain. CAR T cell–based therapies have recently been approved for the treatment of B cell acute lymphoblastic leukemia, diffuse large B cell lymphoma, follicular lymphoma, mantle cell lymphoma, and multiple myeloma. CAR T cell therapies have their origins in the HIV field, albeit initial efforts showed only little clinical efficacy (16–20). More recently, anti-HIV CAR T cell approaches have been developed that target the HIV Env protein expressed on HIV-infected cells through an anti-HIV Env-directed scFv and the ectodomain of CD4 with various intracellular domains (21–26). These can be expressed in a single, dual, or triple CAR construct targeting multiple sites on the HIV Env (21–26). However, the relatively low abundance of HIV-infected cells during the chronic stage and the even lower amount during ART compared with that in certain blood cancers as well as competition with the scFv-based CAR along with anti-CAR antibody responses with de novo antiviral humoral immune response for binding sites on the HIV env have emerged as constraints for a successful anti-HIV CAR T cell approach (27, 28).

Here, we tried to overcome these obstacles by targeting PD-1 as a cellular marker for HIV persistence and replication. Our hypothesis was that there would be a selective increase in PD-1^+^ cells in SIV-infected rhesus macaques (RMs). Moreover, we tested to determine whether CAR T cells could enter the germinal center (GC) and eliminate PD-1–expressing CD4^+^ TFH cells, where latent SIV/HIV infection often occurs. We developed an anti–PD-1 CAR with low tonic signaling that efficiently kills PD-1–expressing cells and can inhibit SIVmac239 infection in vitro. In vivo, in both SIV-naive and SIVmac239-infected RMs on ART, anti–PD-1 CAR T cells expanded in blood and tissues, gained access to immune-privileged sites such as B cell follicles, and depleted PD-1^+^ CD4^+^ T cells as well as strongly reducing numbers of SIV RNA^+^ cells in GCs. To our knowledge, this is the first time such a finding has been described. Importantly, anti–PD-1 CAR T cells persisted without signs of exhaustion, as they did not express detectable cell-surface PD-1, likely because of an interaction in cis with the anti–PD-1 CAR. However, the concomitant destruction of PD-1^+^ memory CD8^+^ T cells exacerbated plasma viral load levels. Notable, SIV replication was shifted to PD-1^–^ cells in the T cell zone in lymphoid tissues. Persistence of the anti–PD-1 CAR T cells coincided with extended lymphopenia and accelerated SIV disease progression, the latter likely due to profound, persistent depletion of both the CD4^+^ and CD8^+^ memory T cell compartment. Collectively, these data indicate that depletion of SIV cellular reservoirs is possible through destruction of anti–PD-1 CAR T cells. However, anti–PD-1 CAR T cell approaches for HIV treatment require additional specificity switches to limit unwanted off-target effects, such as depletion of memory CD8^+^ T cells, for clinical benefits.

## Results

### The anti–PD-1 CAR signals specifically in the presence of PD-1^+^ cells.

To target PD-1 with a CAR, we engineered the anti–PD-1 antibody pembrolizumab, an immune checkpoint inhibitor therapeutic, into the binding domain of a CAR. Mechanistically, pembrolizumab binds to PD-1 and thus shields PD-1–expressing T cells from the interaction with programmed death-ligand 1 (PD-L1), which is commonly expressed on cancer cells to suppress antitumor T cell responses. In contrast, an anti–PD-1 CAR would work through eliminating PD-1^+^ cells.

Six pembrolizumab-derived anti–PD-1 CARs were engineered with 2 different heavy (VH) and light (VL) chain orientations and an IgG4 Fc–derived hinge region of various lengths (a short [S], a medium [M], and a long [L] linker; 12–223 amino acids in length) (Supplemental Figure 1A; supplemental material available online with this article; https://doi.org/10.1172/JCI169309DS1). These were expressed upstream of a truncated version of epidermal growth factor receptor (EGFRt) for cell marking (29) and a c46 fusion inhibitor polypeptide that confers gene protection of CD4^+^ T cells against HIV/SIV infection (28) (Supplemental Figure 1A). We used transduced Jurkat cells to characterize the 6 anti-PD1 CAR constructs. Binding properties and accessibility of the anti–PD-1 CAR paratope differed by scFv orientation, with the VH VL orientation showing higher binding to a PD-1 Fc probe compared with the VL VH orientation (VH VL > VL VH). Similarly, increased binding of the anti–PD-1 CARs to PD-1 was noted with a longer linker compared with a medium or short linker when considering the same scFV orientation (L > M > S), whereas EGFRt expression was similar between constructs (Supplemental Figure 1B and Supplemental Figure 2, A–C).

**Figure 1:**
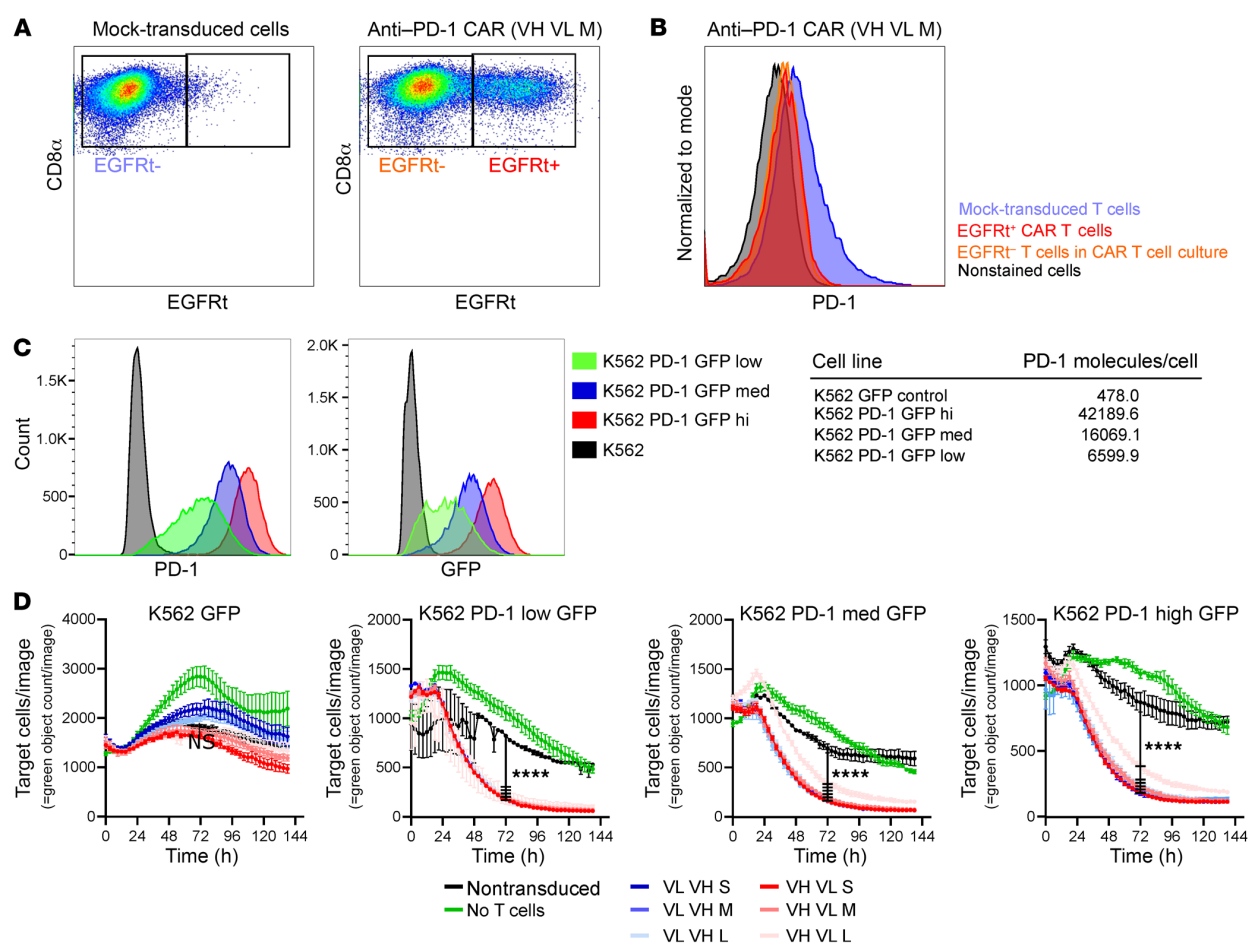
Primary anti–PD-1 CAR T cells do not express PD-1 and kill PD-1–expressing cells. RM CD8^+^ T cells transduced with the 6 anti–PD-1 CAR and analyzed by flow cytometry express EGFRt as a surrogate marker for the CAR (**A**). Gating on EGFRt^+^ and EGFRt^–^ cells in the culture revealed a loss of PD-1 expression on CAR T and bystander cells in CAR T cells in comparison with nontransduced T cells (**B**). PD-1^+^ target cells were generated by transduction of K562 cells with a PD-1 GFP fusion protein–expressing lentivirus followed by sorting for different levels of PD-1 cell-surface expression. PD-1 cell–surface expression was quantified with Quantibrite beads and anti–PD-1 antibody EH12 2H7 (**C**). Anti–PD-1 CAR T cells kill PD-1^+^ target cells. Primary anti–PD-1 CAR CD8^+^ T cells or nontransduced CD8^+^ T cells were cocultured with K562 PD1 GFP with low, medium, and high PD-1 expression or K562-GFP control target cells at a 1:1 ratio. Cytotoxicity was measured by reduction of GFP^+^ cells using the IncuCyte Live-Cell Imaging System. Average cytotoxicity ± SEM of 1 representative experiment is shown (*n* = 3). Statistics were analyzed using 2-way ANOVA with Tukey’s multiple-comparisons test at each time point. Results for 72 hours are reported. ****P < 0.0001 (**D**).

Upregulation of the transcription factor Nur77 downstream of the endogenous TCR signaling chain has been used to study tonic signaling in the absence of antigen, a predictor for poor in vivo performance, and antigen-specific signaling in CAR high-throughput screens (30–32). Baseline Nur77 expression was low for the 6 anti–PD-1 CAR variants and comparable to another CAR based on the broadly neutralizing antibody CAP256-VRC26.25 (CAPCAR) and nontransduced Jurkat cells (28, 33), whereas stimulation through the endogenous TCR induced expression of Nur77 (Supplemental Figure 1C). Antigen-specific signaling of the 6 anti–PD-1 CAR or the CAPCAR was tested by coculture of the CAR Jurkat cell lines with K562 PD-1 GFP-high or K562-GFP as control cells. Nur77 expression was upregulated in all 6 anti–PD-1 CAR Jurkat cell lines, but not in CAPCAR Jurkat cells, and the nontransduced Jurkat cells in the presence of the coculture of K562 PD-1 GFP-high (Supplemental Figure 1D). In contrast, Nur77 expression in the anti–PD-1 CAR and control cell lines remained at baseline in the presence of K562-GFP control cells (Figure 1D).

Combined, these data indicate that the 6 anti–PD-1 CAR constructs do not show tonic signaling and that the scFv orientation and extracellular linker length affect the paratope accessibility of the anti–PD-1 CAR. Antigen-specific signaling appeared not to be affected by the scFv orientation or extracellular linker length.

### Primary anti–PD-1 CAR T cells efficiently kill PD-1–expressing cells and do not express PD-1.

To test the anti–PD-1 CAR in primary T cells, RM CD8^+^ T cells were transduced with the 6 anti–PD-1 CAR variants. We detected the EGFRt tag as a surrogate marker for CAR expression for the 6 constructs (Figure 1A and Supplemental Figure 3). CARs being directed against antigens expressed in T cells can at times lead to fratricide and hamper CAR T cell production (34). PD-1 expression was decreased for EGFRt^+^ and EGFRt^–^ T cells in transduced cultures in comparison with mock-transduced T cells (Figure 1B and Supplemental Figure 3), compatible with ongoing killing of PD-1^+^ nontransduced T cells through anti–PD-1 CAR T cells. Pembrolizumab reportedly can shield PD-1 from detection with the anti–PD-1 antibody EH12.2H7 by flow cytometry (35). To determine whether pembrolizumab-based scFv binding to PD-1 on the cell surface in cis may explain low PD-1 levels on anti–PD-1 CAR T cells, Jurkat Nur77-NeonGreen reporter cells were transduced with the VH VL S anti–PD-1 CAR or 3 CAR mutants, which were defective for PD-1 binding (anti–PD-1 CAR BD) by mutation of amino acids implicated in the interaction of pembrolizumab and PD-1 (36), deletion of the CD3z signaling domain (anti–PD-1 CAR ΔCD3z), or a combination of both (anti–PD-1 CAR BD ΔCD3z). The anti–PD-1 CAR ΔCD3z and anti–PD-1 CAR BD ΔCD3z constructs were used to rule out a potential feed-forward loop of PD-1 interaction with the CAR, resulting in activation and upregulation of PD-1. Only the original anti–PD-1 VH VL S CAR and the anti–PD-1 CAR ΔCD3z showed binding of PD-1 Fc, whereas binding was ablated for anti–PD-1 CAR BD and anti–PD-1 CAR BD ΔCD3z variants (Supplemental Figure 4A). None of the cell lines expressed PD-1 (Supplemental Figure 4B). To validate the loss of PD-1 sensing, Jurkat Nur77-NeonGreen reporter cells expressing the original VH VL S CAR or the altered constructs were cocultured with the PD-1^+^ acute lymphoblastic leukemia cell line Molt4. Only the original VH VL S CAR upregulated Nur77-NeonGreen expression as a surrogate marker for CAR signaling (Supplemental Figure 4C), indicating that the binding- and signaling-deficient receptors do not signal in the presence of PD-1.

To further study the interaction of the anti–PD-1 CAR with PD-1, we used Molt4 cells, which express high levels of PD-1 constitutively (Supplemental Figure 4D). Expression of the anti–PD-1 CAR and the 3 defective variants markedly decreased detectable cell-surface PD-1 in the Molt4 cell lines, which expressed binding-competent anti–PD-1 CAR (anti–PD-1 CAR and the anti–PD-1 CAR ΔCD3z), whereas PD-1 expression was unchanged in the Molt4 cells expressing the binding-deficient receptors (anti–PD-1 CAR BD and anti–PD-1 CAR BD ΔCD3z) (Supplemental Figure 4D). Interestingly, the CAR paratope was still detectable in the cell lines expressing the binding-competent anti–PD-1 CAR variants (anti–PD-1 CAR and the anti–PD-1 CAR ΔCD3z), indicating that the CAR paratope was not saturated through the cell-surface PD-1 and remained functional (Supplemental Figure 4E). To further address the subcellular localization of PD-1 after anti–PD-1 CAR engagement, immunofluorescence staining for PD-1 on the Molt4 cell line expressing different versions of the anti–PD-1 CAR was performed and showed that PD-1 is still detectable in close proximity to the cell surface (data not shown). This indicates that PD-1 and anti–PD-1 CAR interaction results in shielding of PD-1 and prevents its detection by flow cytometry.

To assess whether the killing potential of the 6 receptor variants is dependent on PD-1 expression, we transduced K562 cells with lentiviral vector to express a PD-1 GFP fusion protein and generated 3 cell lines coined K562 PD-1 GFP-low, -medium, and -high with differential PD-1 expression. Titration of PD-1 with mouse monoclonal antibody EH12.2H7, which shares the binding site with pembrolizumab and nivolumab (35), indicated that the 3 K562 PD-1 GFP lines expressed between approximately 6,000 and 40,000 PD-1 molecules per cell (Figure 1C).

To assess killing, RM anti–PD-1 CAR CD8^+^ T cells after positive selection for EGFRt were cocultured with K562 cells expressing either GFP or different levels of PD-1 GFP chimeric protein. Inhibition of proliferation of GFP^+^ cells was used as a surrogate marker for cell killing and was followed over time by life cell imaging (Figure 1D and Supplemental Figure 5). Independently of scFv orientation and linker length of the CAR as well as PD-1 expression levels on target cells, the anti–PD-1 CAR T cells efficiently killed and significantly inhibited outgrowth of K562 PD-1 GFP-low, -medium, and -high cells and failed to control K562-GFP cells in the absence of ectopic expression of PD-1 (Figure 1D and Supplemental Figure 5). Combined, these data indicate that the 6 anti–PD-1 CAR constructs may interact with PD-1 in cis with little effect on CAR T cell function and that the 6 anti–PD-1 CAR constructs kill PD-1–expressing cells to a similar degree within the range of our assays. Based on this, we decided to continue with the VH VL S anti–PD-1 CAR construct alone.

### RM anti–PD-1 CAR T cells kill SIV-infected cells in vitro.

**Figure 2:**
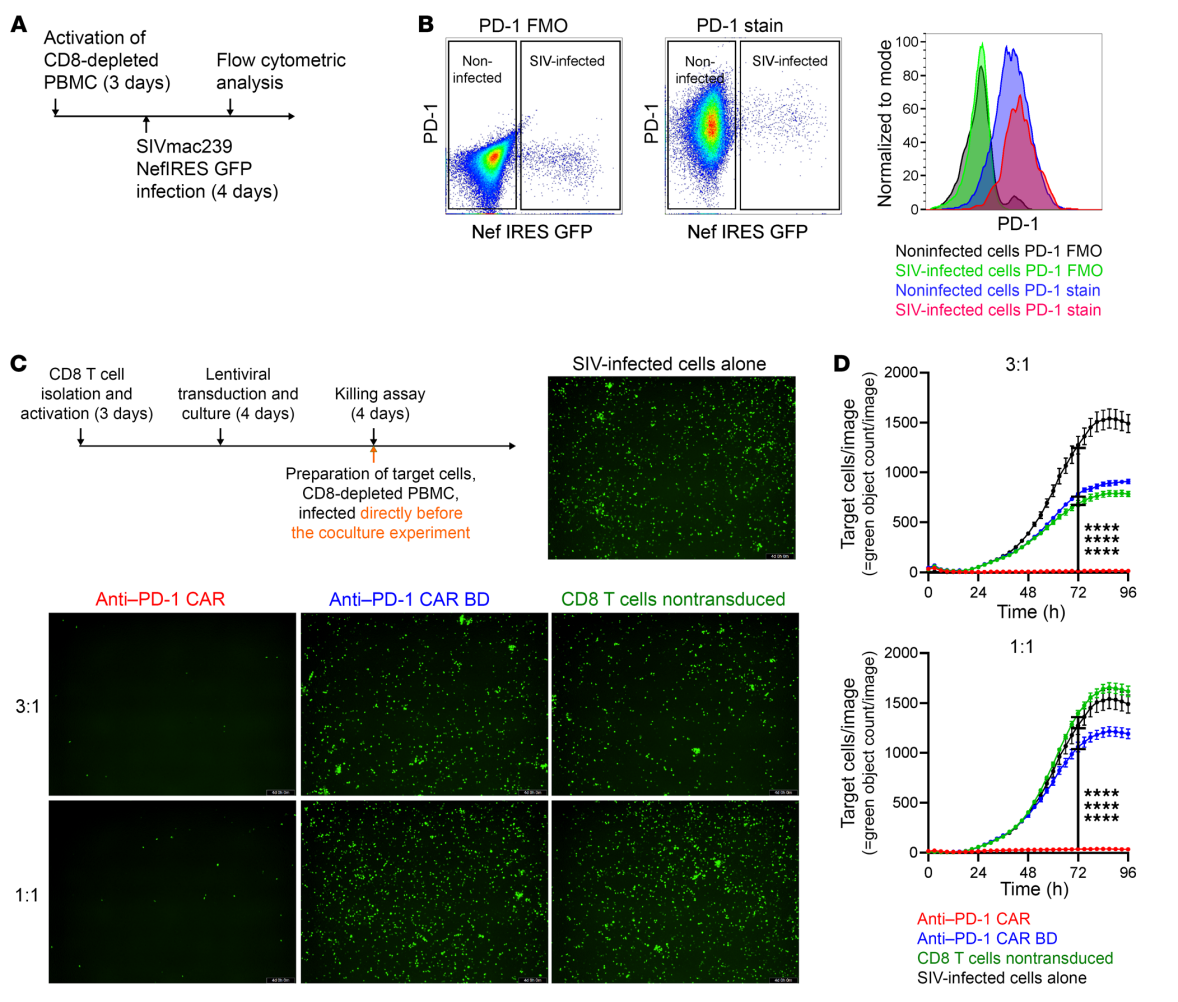
Anti–PD-1 CAR T cells attenuate SIV infection in vitro. Schematic of the production of the infection of CD8-depleted PBMCs with SIVmac239 NefIRESGFP (**A**). PD-1 expression on infected and noninfected cells after 4 days of infection (*n* = 3). Shown are fluorescence minus one (FMO) controls for PD-1 and full staining samples (**B**). Anti–PD-1 CAR T cells prevent viral outgrowth. Schematic showing the time line of preparation of effector cells and autologous CD4^+^ T cells as target cells infected with SIVmac239 NefIRESGFP directly before the experiment. Cytotoxicity was measured by reduction of GFP^+^ cells using the IncuCyte Live Cell Imaging System. Representative images at the end of the 96 hours of coculture with effector cells at the indicated E:T ratio (*n* = 3) (**C**). Original magnification, ×10. Quantification of GFP^+^ cells in the images acquired over 96 hours in the killing assay described in **C** (**D**). Average cytotoxicity ± SEM of 1 representative experiment is shown. Statistics were analyzed using 2-way ANOVA with Tukey’s multiple-comparisons test at each time point. Results for 72 hours are reported. ****P < 0.0001.

We tested to determine whether anti–PD-1 CAR T cells can attenuate viral outgrowth in vitro, as PD-1^+^ CD4 T cells are a major compartment for HIV and SIV persistence and replication on and off ART (5, 9, 14). We initially evaluated whether SIVmac239 preferentially replicates in cells that express PD-1 in an in vitro system. To this aim, activated CD8-depleted PBMCs were infected with the SIVmac239 NefIRESGFP virus strain, which expresses GFP downstream of Nef through an internal ribosome entry site (IRES) sequence (37). PD-1 expression on GFP^+^ cells was assessed after 4 days (Figure 2A). Infected GFP^+^ cells exhibited a higher MFI for PD-1 than total noninfected cells. Interestingly, the PD-1 expression levels on PD-1^hi^ cells did not differ between infected and noninfected cells (Figure 2B). Of note, in our in vitro model, more than 95% of CD4^+^ T cells differentiated into an effector-memory phenotype based on CD28^lo^CD95^hi^ expression by day 4 after infection and day 7 after activation, respectively (data not shown). To determine whether anti–PD-1 CAR T cells can attenuate SIV replication, we used a live-cell imaging killing assay with autologous cells to prepare the CAR T cells from CD8^+^ T cells and cocultured them with autologous SIVmac239 NefIRESGFP–infected CD8-depleted PBMCs as target cells for 96 hours. CD8^+^ T cells expressing the anti–PD-1 CAR, the binding-deficient anti–PD-1 CAR BD, or nontransduced CD8^+^ T cells were used as effector cells. The target cells were infected either directly or 4 days prior to the start of the coculture experiment to determine whether the anti–PD-1 CAR can prevent viral outgrowth and ongoing infection, respectively.

When the target cells were infected for 4 days prior to the killing assay, anti–PD-1 CAR T cells significantly reduced SIV-infected cells at an effector/target (E:T) ratio of 3:1 and 1:1 compared with control effector cells and infected target cells alone (Supplemental Figure 6, A and B). In contrast, nontransduced CD8^+^ T cells and anti–PD-1 CAR BD cells failed to attenuate viral infection in the culture (Supplemental Figure 6, A and B), and viral outgrowth kinetics were similar to SIV-infected cells alone (Supplemental Figure 6B). Similarly, when the target cells were infected immediately before the killing assay, anti–PD-1 CAR T cells significantly reduced SIV-infected cells at an E:T ratio of 3:1 and 1:1 compared with control effector cells and infected target cells alone (Figure 2, C and D). In contrast, nontransduced CD8^+^ T cells and anti–PD-1 CAR BD cells failed to attenuate viral infection in the culture (Figure 2, C and D) and viral outgrowth kinetics were similar to SIV-infected cells alone (Figure 2D). Combined, these data indicate that anti–PD-1 CAR T cells can attenuate SIVmac239 viral outgrowth in vitro.

### PD-1^+^ cell aplasia following adoptive transfer and in vivo expansion of anti–PD-1 CAR T cells.

A key feature of HIV infection is its ability to persist in immunologically privileged sites, such as LN GCs. We utilized the nonhuman primate (NHP) model to evaluate whether our anti–PD-1 CAR T cells could traffic into GC and reduce SIV-infected PD-1^+^ T cells in vivo. To that end, we performed a series of adoptive transfer experiments in both SIV-naive and SIVmac239-infected RMs on ART. In the first study, 2 animals with no SIV infection received CAR T cells expressing the anti–PD-1 CAR EGFRt c46 construct. In the second study, 4 SIVmac239-infected RMs were infused with the anti–PD-1 CAR EGFRt c46 construct (*n* = 2) or with an anti–PD-1 CAR EGFRt c46 CXCR5 construct (*n* = 2) (Supplemental Figure 7A), which differentially expressed CXCR5 in vitro (Supplemental Figure 7B). We used a previously described CAR T cell production protocol that aims to achieve a 1:1 ratio of CD4^+^ and CD8^+^ CAR T cells (27, 28). Of note, human and RM PD-1 are 96% identical in protein sequences, and the amino acids implicated in the interaction with pembrolizumab are 100% conserved between human and RM (Supplemental Figure 8) (36), which may indicate similar anti–PD-1 CAR affinity and signaling strength in response to human and RM PD-1.

**Figure 3:**
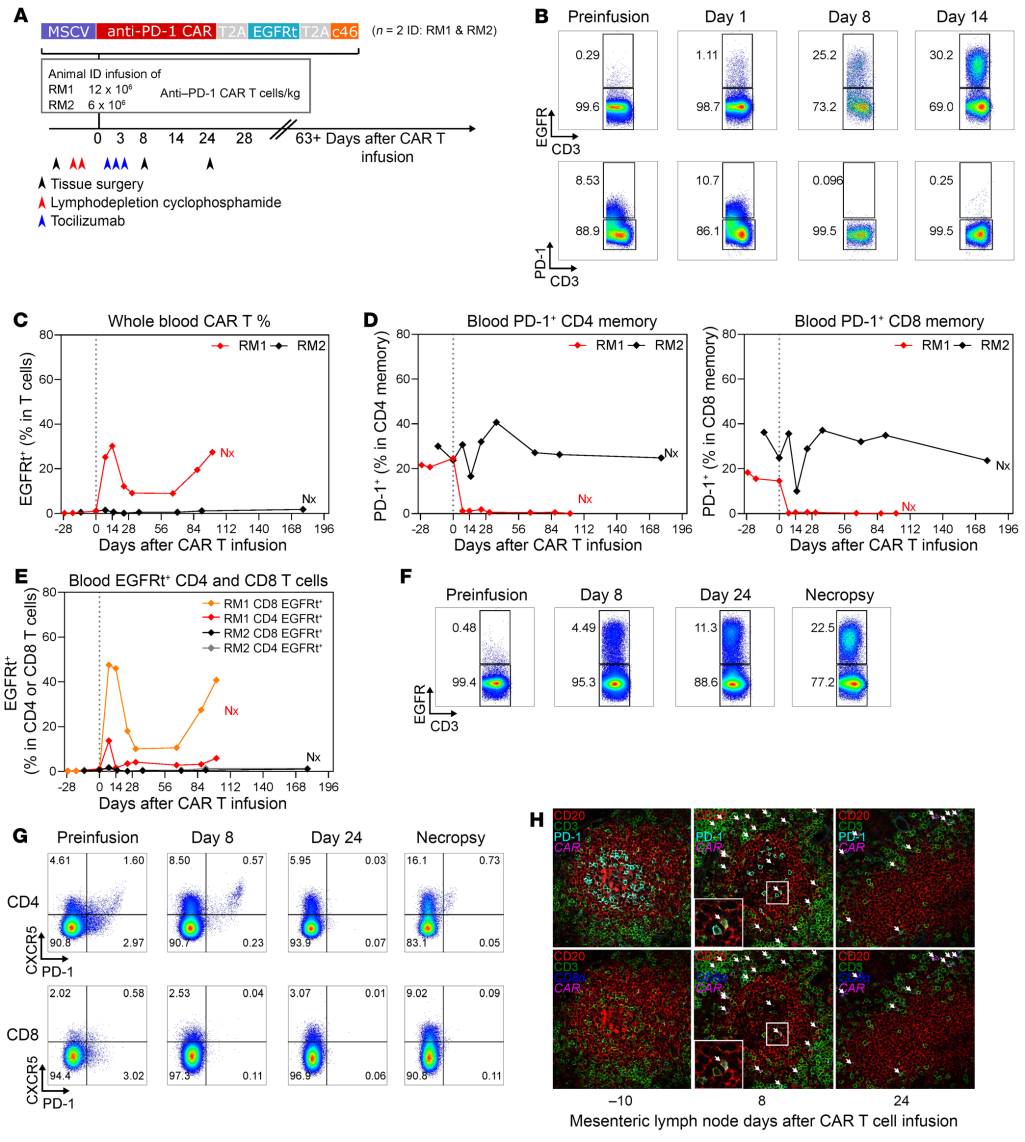
Anti–PD-1 CAR T cells expand in vivo and deplete PD-1^+^ CD4^+^ and CD8^+^ T cells in an SIV-naive RM. Schematic of the in vivo animal study in SIV-naive RMs (*n* = 2). Dates for tissue surgery, lymphodepletion, and tocilizumab treatment are noted (**A**). EGFRt and PD-1 cell marking in total CD3^+^ T cells in vivo at days 8 and 14 indicates robust expansion of anti-PD1 CAR T cells in RM1 (**B**). Longitudinal PBMC sampling in RM1 and RM2 for percentages of total CAR T cells in CD3^+^ T cells (**C**) and PD-1^+^ in CD4^+^ and CD8^+^ T cells (**D**). Nx, necropsy. Flow cytometric analysis of EGFR^+^ CD4^+^ or CD8^+^ CAR T cells in peripheral blood in RM1 (**E**). Expansion of CAR T cells in Peri.LN of RM1 (**F**). Depletion of PD-1–expressing TFH cells in LNs was assessed by flow cytometry (**G**). Combined multicolor IHC and RNA FISH for CD3 (green), CD20 (red), PD-1, CD8α RNA (blue), and CAR RNA (cyan) on B cell follicles of mes.LNs on days –10, 8, and 24 relative to infusion. White arrows indicate the location of anti–PD-1 CAR CD8^+^ T cells (**H**). Original magnification, ×40.

The schema used for adoptive transfer of anti–PD-1 CAR T cells into 2 SIV-naive RMs is outlined in Figure 3A. Adoptive transfer of anti–PD-1 CAR T cells into 2 SIV-naive RMs was performed at a dose of 6 to 12 × 10^6^ cells/kg using cells expressing the anti–PD-1 CAR-EGFRt-C46 construct (animal ID: RM2 and RM1; Supplemental Table 1). In vitro killing assays to assess functionality of the infusion product showed potent killing in the presence of PD-1^+^ KG cells at an E:T ratio of 3:1 for both RM2 and RM1 (Supplemental Figure 9, A and B); however, efficient killing at a lower E:T ratio indicated that the RM1 infusion product (Supplemental Figure 9A) was more potent than the RM2 infusion product (Supplemental Figure 9B).

Lymphodepletion was performed 3 and 4 days prior to CAR T cell infusion (Figure 3A), and proliferation after adoptive transfer was successful in the animal receiving the higher dose (ID: RM1), with a bimodal CAR T cell expansion peaking at day 14 with 30.2% EGFRt^+^ cells of total CD3, an intermittent phase of lower frequency, and a second peak at day 100 (Supplemental Table 1 and Figure 3, B and C). For the second RM (ID: RM2), we observed more moderate CAR T cell expansion (up to 6.49% EGFRt^+^ cells of total CD3) peaking at day 3 with lack of CAR T cell persistence (Figure 3C). Depletion of total PD-1^+^ memory CD4^+^ and CD8^+^ T cells based on CD28^+^CD95^+^ and CD28^–^CD95^+^ expression was maintained for 100 days in RM1, whereas RM2 showed no sign of depletion (Figure 3D). The majority of expanding and persisting anti–PD-1 CAR T cells were CD8^+^ (Figure 3E). Anti–PD-1 CAR T cell expansion was detectable in peripheral LN (peri.LN) tissues of RM1 on days 8, 24, and 100 (necropsy time point) after infusion by flow cytometry (Figure 3F) and occurred concomitantly with depletion of memory PD-1^+^ CD8^+^ and PD-1^+^ CD4^+^ as well as TFH cells based on coexpression of PD-1 and CXCR5 in peri.LN and mesenteric LN (mes.LN) (Figure 3G and Supplemental Figure 10A). No sign of TFH depletion was noted in RM2 at necropsy (Supplemental Figure 10A). We used multicolor RNA FISH and immunofluorescence microscopy to confirm access of anti–PD-1 CAR CD8^+^ T cells to B cell follicles along with depletion of TFH cells. Intrafollicular PD-1 expression markedly decreased at days 8 and 24 after infusion and was accompanied by the presence of CD8a^+^ CAR RNA^+^ T cells within the perimeter (Figure 3H and Supplemental Figure 10B). Combined, these data indicate that anti–PD-1 CAR T cells can expand in blood and tissues in SIV-naive RMs and deplete PD-1^+^ CD8^+^ and CD4^+^ memory T cells, including TFH cells. The ability to successfully transfer functional T cells and the evidence of trafficking into GC with expansion of CAR T cells after infusion in the initial pilot led to our experiments with SIV-infected RMs.

**Figure 4:**
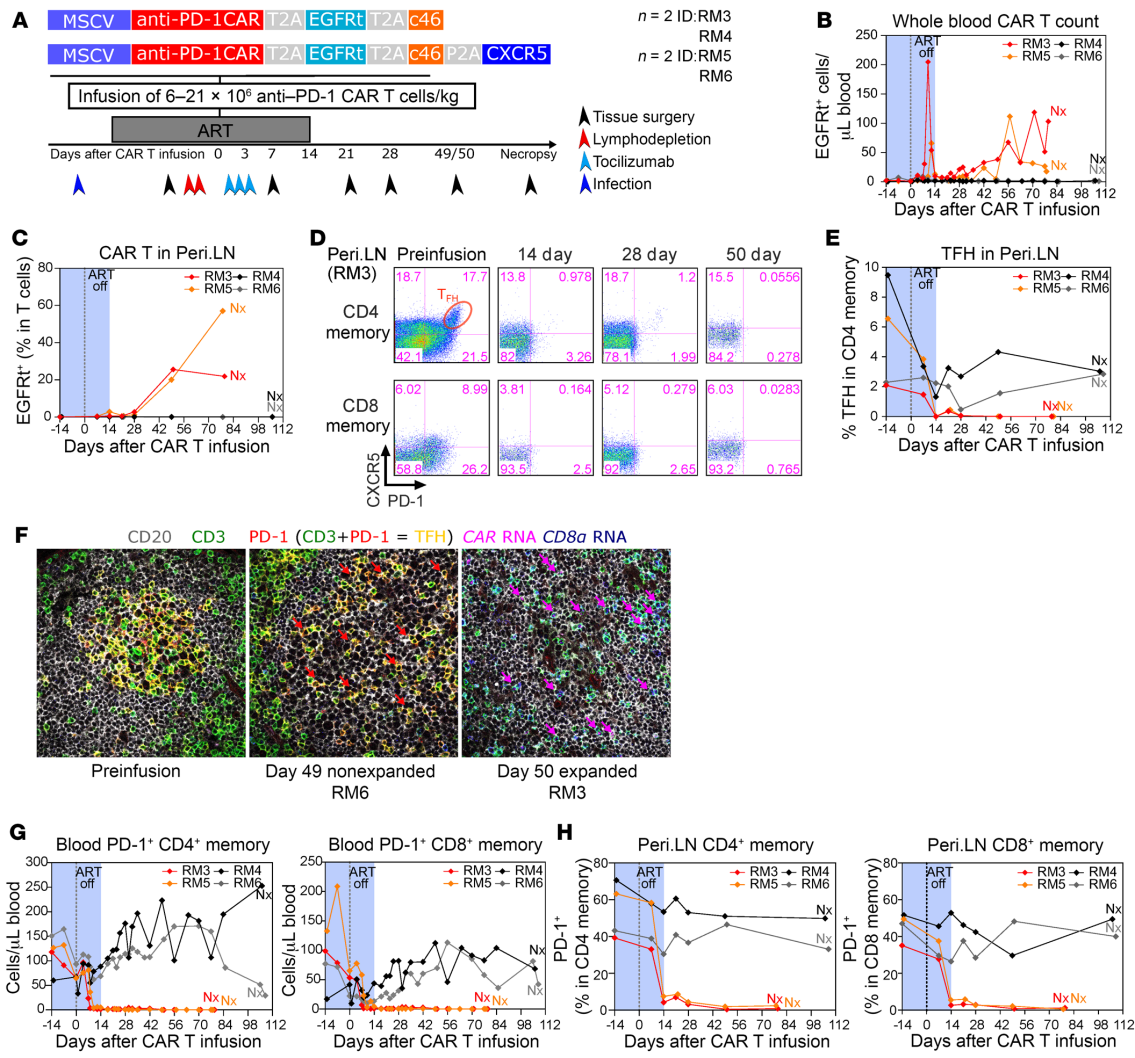
Anti–PD-1 CAR T cells deplete PD-1^+^ T cells in SIV-infected RMs on ART. Schematic of the in vivo animal study (*n* = 4). Dates for tissue surgery, lymphodepletion, duration of ART, and tocilizumab treatment are noted (**A**). Absolute count of EGFRt-expressing CD3^+^ T cells in peripheral blood for the duration of the study (**B**). Longitudinal EGFRt-expressing CD3^+^ T cells in peri.LN. (**C**). Flow cytometric analysis of PD-1 and CXCR5 expression on CD4^+^ and CD8^+^ total memory T cells in Peri.LN from RM3 (**D**). Frequency of TFH cells in CD4^+^ total memory T cells (**E**). Combined immunofluorescence and RNA FISH staining on LN tissue section for CD20 (gray), CD3 (green), PD-1 (red), CD8α RNA (blue), and CAR RNA (magenta) (**F**). Magenta arrows point to intrafollicular CD8^+^ CAR T cells, and red arrows point to residual TFH cells after infusion. TFH cells are characterized by CD3 and PD-1 dual staining within the B cell follicle and appear yellow (**F**). Original magnification, ×40. Absolute count of PD-1^+^ CD4^+^ and CD8^+^ memory T cells in peripheral blood (**G**). PD-1 expression on Peri.LN CD4^+^ memory T cells and CD8^+^ memory T cells (**H**).

The schema used for adoptive transfer of anti–PD-1 CAR T cells into 4 SIV-infected ART-treated RMs is outlined in Figure 4A. The RMs were inoculated with SIVmac239, and ART was initiated 12 days after inoculation. The animals were infused with T cells engineered to express an anti–PD-1 CAR-EGFRt-c46 (*n* = 2, ID RM3 and RM4) or an anti–PD-1 CAR-EGFRt-c46-CXCR5 expression cassette (*n* = 2, ID RM5 and RM6) at least 3 months after ART initiation and released from ART 14 days after CAR T cell infusion (Figure 4A). Removal of ART at 14 days after infusion was based upon the adoptive transfer experiments in SIV-naive RM in which CAR T cell expansion peaked 14 days after infusion. We reasoned that having maximal anti–PD-1 CAR T cell expansion at the time of ART release would allow us to determine the impact of TFH and PD-1^+^ CD4^+^ T cell depletion on SIVmac239 rebound kinetics. The cell dosage ranged between 6 and 20.8 × 10^6^ CAR T cells/kg, and additional characteristics for each infusion product are shown in Supplemental Table 1. Killing efficacy was assessed for each infusate, which indicated that the infusion product for RM6 (Supplemental Figure 11A) had only little potency in vitro, whereas the infusates for RM4 and RM3 (Supplemental Figure 11, B and C) were potent at killing KG PD-1 cells across different E:T ratios after 72 hours of coculture. The infusion product for RM5 showed statistically significant killing efficacy after 96 hours of coculture (Supplemental Figure 11D). All infusions were well tolerated without occurrence of CAR T cell–related adverse events. No clinical neurotoxicity was observed, in contrast with previous reports with anti-CD20 CAR T cell infusion in RM (38). Anti–PD-1 CAR T cells expanded in peripheral blood of 2 of the 4 animals (RM3 and RM5), with initial peak expansion of 200 and 60 EGFRt^+^ cells/μL blood at days 10 and 14, respectively (Figure 4B, Supplemental Table 1, and Supplemental Figure 12A). Peri.LN trafficking of anti–PD-1 CAR T cells occurred in both animals, with evidence of expansion in blood (Figure 4C and Supplemental Figure 12A). CAR T cells were also detected in spleen, mes.LNs, liver, lung (bronchoalveolar lavage [BAL]), BM, and, to a lesser extent, in the gastrointestinal tract (Supplemental Figure 12B). At necropsy, CAR T cells were detectable by flow cytometry at more than 10% in most lymphoid tissues, liver, BAL, BM, ileum, and perfused brain of RM3 and RM5, but not in the 2 animals with no evidence of postinfusion expansion: RM6 and RM4 (Supplemental Figure 13). Interestingly, CAR T cells were not detectable in jejunum and thymus at necropsy (Supplemental Figure 13). In accordance with our in vitro data, anti–PD-1 CAR T cell infusion products and in vivo–expanded CAR T cells lacked PD-1 expression (Supplemental Figure 14A). Analysis of longitudinal samples indicated that anti–PD-1 CAR T cells had no measurable PD-1 expression at any point throughout the study time course in either the SIV-infected (Supplemental Figure 14B) or SIV-naive RMs (Supplemental Figure 14C).

Depletion of TFH cells, characterized by high coexpression of PD-1 and CXCR5, occurred in animals with CAR T cell expansion in peri.LNs at the time of ART release (Figure 4, D and E) as well as in mes.LNs and the spleen (Supplemental Figure 15A). To confirm trafficking of anti–PD-1 CAR CD8^+^ T cells to B cell follicles as well as CAR T cell–mediated depletion of TFH cells, we used a combined immunofluorescence and RNA FISH assay for *CAR* RNA, CD8α RNA, PD-1, CD20, and CD3. We detected intrafollicular anti–PD-1 CAR CD8^+^ T cells and depletion of TFH cells in GCs of the B cell follicles in day 49/50 tissue sections in the 2 animals with anti–PD-1 CAR T cell expansion (RM3, RM5), but not in the RMs without CAR T cell expansion (RM6, RM4) (Figure 4F and Supplemental Figure 15B). Of note, there appeared to be no difference in B cell follicle access between anti–PD-1 CAR T cells that coexpressed CXCR5 (RM5) and those that did not (RM3) (data not shown). In summary, these studies in SIV-naive and SIV-infected RMs indicate that anti–PD-1 CAR T cells are efficient in accessing immune-privileged sites such as the B cell follicles and can deplete TFH cells, a major site of HIV replication (Supplemental Table 2). Depletion of PD-1^+^ memory CD4^+^ (Supplemental Figure 16) and CD8^+^ T cells (Supplemental Figure 17) occurred also in other secondary lymphoid tissues, the lung, liver, duodenum, and colon. Depletion of peripheral blood memory PD-1^+^ CD4^+^ and CD8^+^ T cells occurred concomitantly with CAR T cell expansion in RM3 and RM5, but not in RM6 and RM4 (Figure 4G). Depletion of memory PD-1^+^ CD4^+^ and CD8^+^ T cells was long-lasting until necropsy (Figure 4G), consistent with persistence of CAR T cell expansion (Figure 4B). Similarly, long-lasting depletion of PD-1^+^ memory CD8^+^ and CD4^+^ T cells was also documented in peri.LNs in animals with CAR T cell expansion (Figure 4H).

### Viral rebound occurs in the absence of TFH cells in extrafollicular T cells.

**Figure 5:**
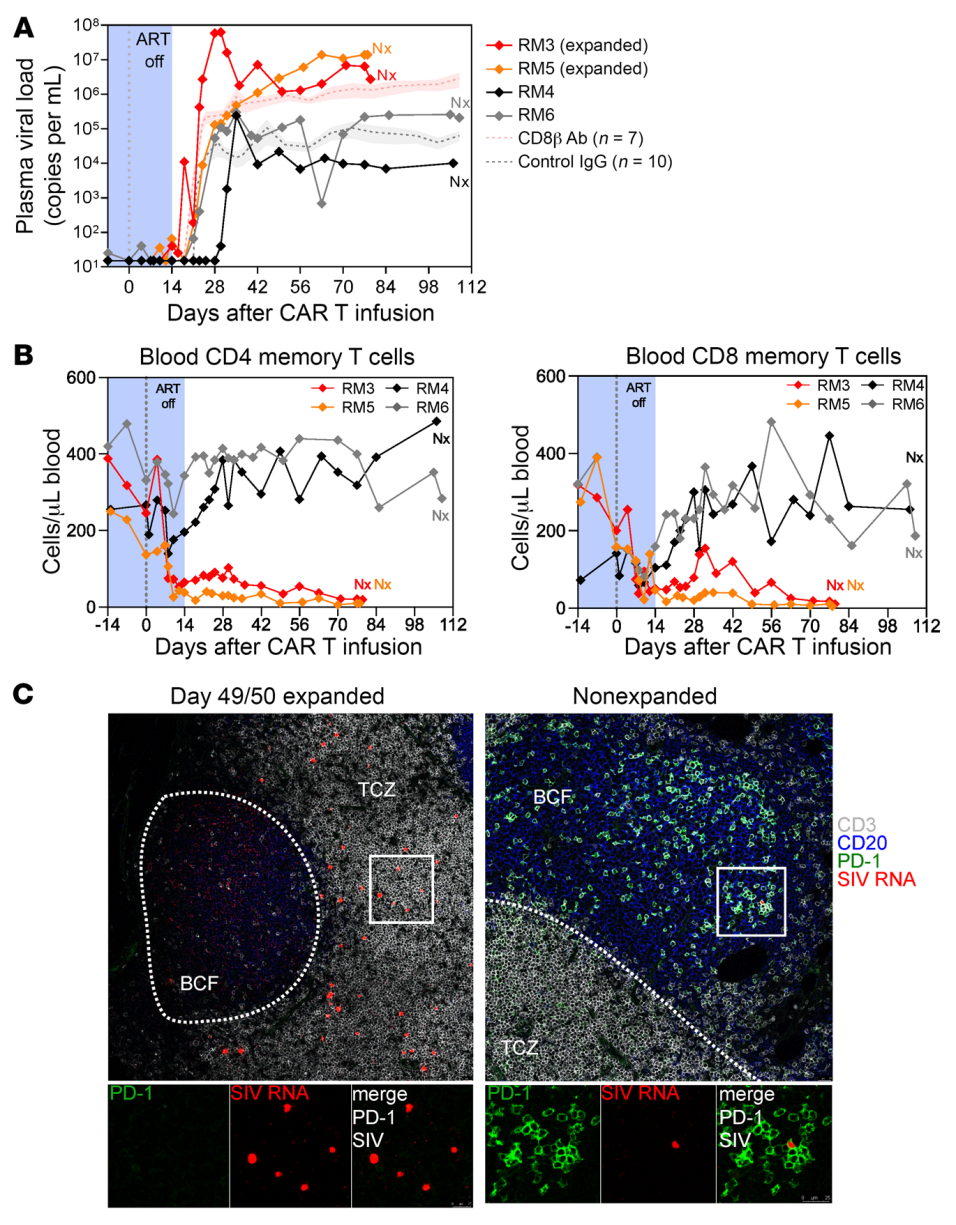
Anti–PD-1 CAR T cell–mediated depletion of TFH cells and PD-1^+^ T cells does not prevent viral recrudescence after removal of ART. Plasma viral load in the 4 animals during anti–PD-1 CAR T cell treatment and after removal of ART. Historical controls are shown for the indicated number of RMs as mean (+SEM) (**A**). Absolute count of CD4^+^ memory and CD8^+^ memory T cells in blood (**B**). Combined immunofluorescence and RNA FISH staining on LN tissue section for CD3 (gray), CD20 (blue), PD-1 (green), and SIV RNA (red). The white line demarcates the border between the T cell zone (TCZ) and the B cell follicle (BCF) (**C**). Original magnification, ×40.

PD-1^+^ CD4^+^ T cells and TFH cells play a central role in HIV and SIV infection and persistence (5, 9) and were successfully depleted by anti–PD-1 CAR T cells. However, viral rebound after removal of ART occurred in all 4 animals independently of CAR T cell expansion and depletion of PD-1^+^ TFH cells. In addition, plasma viral load was notably higher (~2 log) in RM3 and RM5 (Figure 5A), the animals with CAR T cell expansion. This loss of viral control in the RMs with anti–PD-1 CAR T cell expansion was comparable to historic data from a previous study in which CD8^+^ T cells were depleted with a rhesusized anti-CD8β monoclonal antibody at the time of ART removal (39). Accordingly, a substantial depletion of memory CD4^+^ and CD8^+^ T cells was observed in the RMs with CAR T cell expansion (Figure 5B), indicating that potentially the loss of SIV-specific memory CD8^+^ T cells in the extrafollicular areas led to the higher plasma viral load levels. Precluding the increased high viral load in the 2 RM with anti–PD-1 CAR T cell expansion was a 42.8- and 13.2-fold increase in cell-associated viral RNA levels measured in PBMCs and LNs in the 2 RM with anti–PD-1 CAR T cell expansion (versus 2.5- and 0.9-fold in the 2 RM without anti–PD-1 CAR T cell expansion) on day 14 compared with a preinfusion time point (Supplemental Figure 18). In contrast, cell-associated DNA levels remained stable in the same time frame (Supplemental Figure 18).

**Figure 6:**
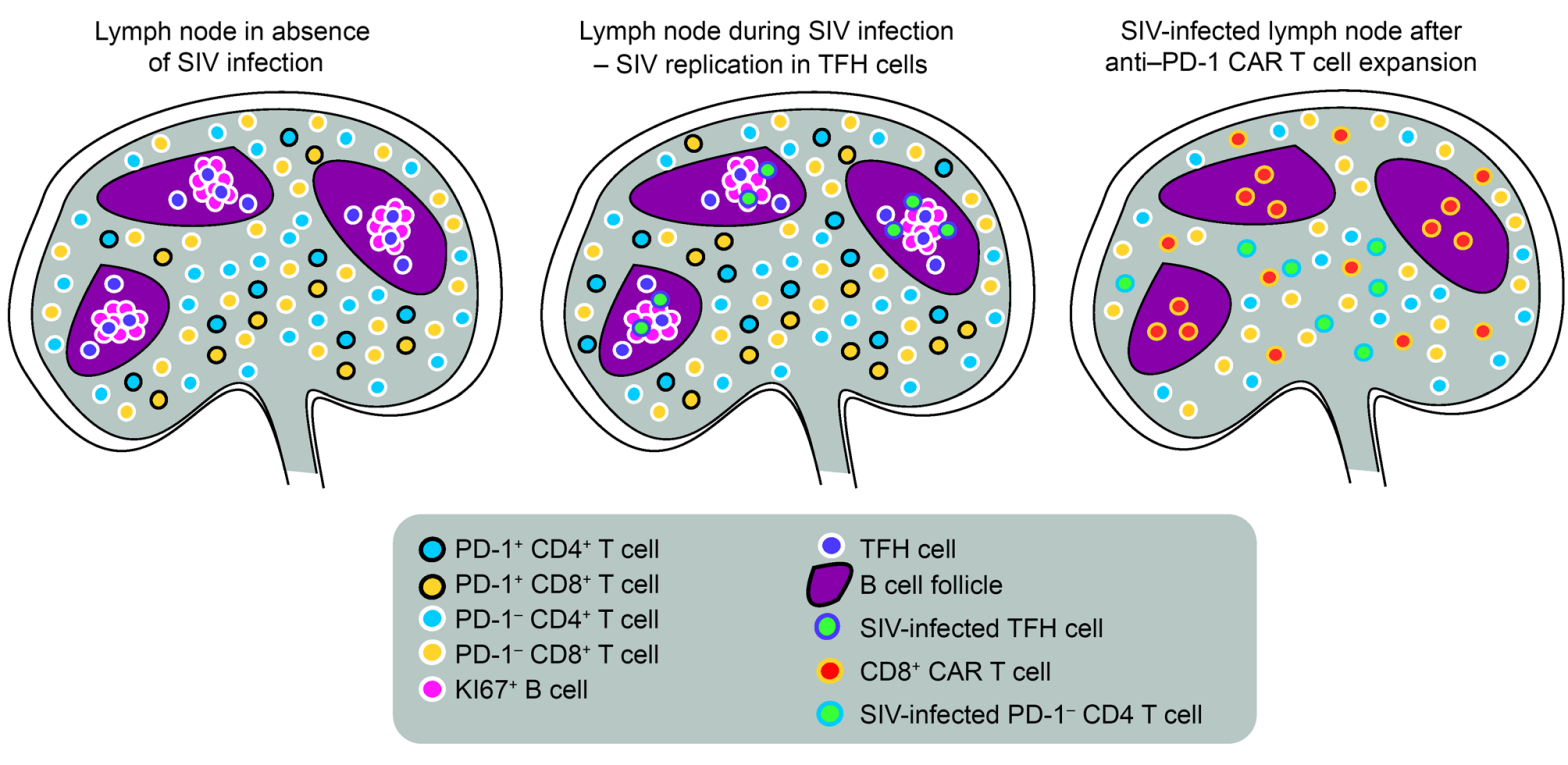
Effects of anti–PD-1 CAR T cells in SIV-infected RMs. At steady state, TFH cells are localized in B cell follicles, where they support B cell responses. Extrafollicular PD-1–expressing T cells are part of the memory compartment or have been recently exposed to antigen. During lentiviral infection, there is an increase of TFH cells, extrafollicular PD-1^+^ CD4^+^ and CD8^+^ T cells, and viral replication occurring predominantly in TFH cells. During successful expansion, anti–PD-1 CAR T cells enter lymphoid tissues and kill extrafollicular PD-1^+^ T cells as well as TFH cells in the follicles. This results in relatively lower viral replication in the follicles due to depletion of TFH cells and increases viral replication in the extrafollicular T cell zone where it occurs in PD-1 CD4^+^ T cells. SIV-specific T cells are likely part of the extrafollicular PD-1^+^ T cell population and are lost due to depletion of anti–PD-1 CAR T cells. Over time, this leads to anti–PD-1 CAR T cell–mediated immunodeficiency.

Using combined immunofluorescence and RNA FISH for SIV RNA, PD-1, CD20, and CD3, we detected SIV^+^ PD-1 T cells in the perifollicular zone in day 49/50 tissue section in the 2 animals with anti–PD-1 CAR T cell expansion (RM3, RM5), but not in the ones that failed to expand (RM6, RM4) (Figure 5C). Of note, we did not detect SIV RNA^+^ cells in lymphoid tissues of RM4 at this time point (data not shown), which is also reflected in the low plasma viral load after ART removal (Figure 5A). Intrafollicular SIV RNA^+^ cells were largely absent in RM3 and RM5 49/50 days relative to CAR T infusion (Figure 5C). However, single viral particles were present on the follicular dendritic cell network, indicating the specificity of the CAR T cells for eradicating PD-1–expressing SIV^+^ cells (Figure 5C). In RM6, which had no CAR T cell expansion, PD-1^+^ SIV RNA^+^ T cells were observed within B cell follicles (Figure 5C and Supplemental Figure 19). These data indicate that anti–PD-1 CAR T cells can successfully expand in SIV-infected RM and deplete TFH as well as PD-1^+^ CD4^+^ and CD8^+^ T cells (Figure 6). Furthermore, depletion of PD-1^+^ CD4^+^ T cells, a major cell compartment that harbors the SIV replication-competent reservoir and an important site for persistent virus replication, at the time of ART removal focused viral replication to the extrafollicular zone, which was associated with plasma viral rebound of SIV. Specific depletion of PD-1^+^ CD4^+^ and CD8^+^ T cells appears to have induced a form of CAR-mediated immunodeficiency with an attenuation of cellular and humoral responses, which likely exacerbated SIV infection.

### Persistence of anti–PD-1 CAR T cells in SIV-naive and SIV-infected RMs mediates an anti–PD-1 CAR T cell–induced immunodeficiency.

The infusion and expansion of anti–PD-1 CAR T cells concomitant with depletion of PD-1^+^ T cells occurred without laboratory or clinical evidence of cytokine release syndrome. No evidence of the neurological symptoms reported with anti-CD19 or anti-CD20 CAR T cells was noted (38, 40). One of the interesting observations of our study was the long-term persistence of the CAR T cells. From 10% to 40% CAR T cells of total T cells in PBMCs were seen at more than 30 days after infusion. This anti–PD-1 CAR expansion and persistence were associated with long-lasting depletion of memory CD4^+^ and CD8^+^ T cells in both SIV-infected animals (ID: RM3 and RM5) (Figure 5B). This immunosuppression was associated with monocytosis, hyperalbuminemia, diarrhea, and anemia (Supplemental Tables 2 and 3). Recovery of total CD4^+^ T cells was also hampered in the SIV-naive animal (ID: RM1) (Supplemental Figure 20A). Interestingly, absolute memory CD8^+^ T cell count remained stable at a higher level in the SIV-naive animal with CAR T cell expansion (ID: RM1: 200 cells/μL ± 60 cells/μL blood) after infusion (Supplemental Figure 20B) than observed in SIV-infected animals (ID: RM3 and RM5) (Figure 5B). RMs RM1, RM3, and RM5 were necropsied on days 100, 79, and 78 after infusion, respectively, due to meeting clinical end points. At necropsy, high frequencies of CAR T cells were seen in lymphoid tissue as well as lung, liver, and brain (Supplemental Figure 13). Anti–PD-1 CAR T cells were not seen in animals that did not show initial CAR T cell proliferation after infusion. TFH cells are an important component of the humoral immune response; therefore, we interrogated how the anti–PD-1 CAR-mediated depletion of TFH cells affected humoral immunity. Proliferating Ki67^+^ B cells are located predominately in GCs within the follicles in lymphoid tissues and are dependent on the presence of TFH cells to provide cytokines for proliferation. However, at necropsy, we observed a loss of Ki67^+^ B cells within the GCs of the SIV-naive RM RM1 with CAR T cell expansion (Supplemental Figure 20C). For the SIV-infected RMs, we performed multicolor immunofluorescence staining for CD3, CD20, and Ki67 to confirm the loss of Ki67^+^ B cells in GCs on days 49/50 after infusion. Anti–PD-1 CAR T cell expansion notably decreased Ki67^+^ B cells, whereas the 2 SIV-infected RMs in which no anti–PD-1 CAR T cell expansion occurred retained fully developed B cell follicles with abundant Ki67^+^ cells in GCs (Supplemental Figure 20D). To assess how the depletion of TFH cells affected memory B cell responses and antibody recall responses, longitudinal serum samples before and after anti–PD-1 CAR T cell infusion were assessed for the presence of anti-SIV neutralizing antibodies. We noted substantial increases in neutralization titers were observed between week 2 and week 5 after infusion, with a 1- to 2-log increase in titers for the 4 RMs (Supplemental Table 3) regardless of anti–PD-1 CAR T cell proliferation, suggesting memory B cell responses are maintained.

Postmortem analysis of the SIV-infected RMs indicated increased atypical leukocyte proliferation in lymphoid tissues and BM as well as most major organs, which was associated with lymphocryptovirus (LCV) RNA^+^ staining in animals with CAR T cell expansion (Supplemental Table 4). LCV is a well-documented etiological agent of atypical non-Hodgkin lymphoma–like lymphocytosis in SIV-infected RMs, and the earlier onset in the absence of immune effector cells such as CD8^+^ T cells has been observed previously (41). These studies indicate that anti–PD-1 CAR T cells can induce T cell–related immunodeficiency in a healthy RM and contribute to total loss of memory T cells, AIDS-related opportunistic infections, and accelerated disease progression in SIV-infected RMs.

## Discussion

We developed an anti–PD-1 CAR to target PD-1–expressing cells and tested its safety profile, expansion, and tissue trafficking in vivo in both SIV-uninfected and ART-treated SIVmac239-infected RMs. Anti–PD-1 CAR T cells expanded successfully in 3 out of 6 RMs; all 3 of these animals, whether SIV infected or not infected, consistently depleted PD-1^+^ T cells, including CD4^+^ TFH cells, in both blood and tissues. This CAR T cell expansion was associated with high-density infiltration of anti–PD-1 CAR T cells in the GCs, subsequent elimination of TFH cells, and marked loss of SIV RNA^+^ cells in the GCs in association with the absence of TFH cells. To our knowledge, this is the first demonstration of successful inhibition of viral replication in this immune-privileged viral sanctuary and offers potential for a conceptual advance in the HIV-cure field. This depletion of CD4^+^ TFH cells was partially achieved as early as day 7 after infusion, a week prior to ART release. CD4^+^ TFH cells were completely ablated by day 14 after infusion, notably the last day of ART, suggesting that release from ART may not be necessary for TFH depletion and elimination of SIV/HIV from this reservoir and that rapid elimination of the anti–PD-1 CAR T cells may be a potential strategy for reducing the effects of their long-term persistence. Interestingly, the anti–PD-1 CAR T cells exhibited prolonged biologically active persistence in vivo. Among the 3 RMs with initial expansion, all exhibited continued expansion of anti–PD-1 CAR T cells in peripheral blood over the 3-month follow-up period, often at high levels. This expansion was associated with widespread depletion of PD-1^+^ T cells and loss of both memory CD4^+^ and CD8^+^ T cells. Loss of the T cell memory compartment by our approach has 2 important implications; first, the anti–PD-1 CAR did not discriminate between higher PD-1 expression on TFH cells and relatively lower PD-1 expression on memory CD4^+^ and CD8^+^ T cells, and second, PD-1 was expressed on all memory CD4^+^ and CD8^+^ T cells during their maturation from naive T cells. Elimination of the memory T cell pool was associated with clinical immunodeficiency.

In SIV-infected RMs that showed CAR T cell expansion, SIVmac239 replication increased despite near complete depletion of PD-1^+^ CD4^+^ T cells at the time of ART removal; post-ART plasma viral load set points were about 2 logs higher relative to RMs without CAR T cell expansion. This expansion of viral RNA in plasma was associated with a remarkable increase in extrafollicular SIV-infected PD-1 CD4^+^ T cells, a pattern seen in chronic-phase viremic SIVmac239-infected RMs that progress to AIDS rapidly, but not in elite controller RMs (5). This further illustrates the importance of antiviral activity of the T lymphocyte population in these tissue sites. Future studies should focus on more detailed kinetics of this reactivation of lentivirus infection in these anatomic sites and the role and mechanism that endogenous immune responses play in viral containment during both suppressive therapy and release. Long-term persistence of the anti–PD-1 CAR T cells appeared to accelerate disease progression in the SIV-infected animals, potentially due to diminished SIV-specific effector CD4^+^ and CD8^+^ T cell responses (Figure 6). Interestingly, memory CD8^+^ T cell depletion was less pronounced in the SIV-uninfected RMs; however, loss of memory CD4^+^ T cells was demonstrated. Thus, while our primary hypothesis that anti–PD-1 CAR T cells could selectively abrogate TFH cells containing SIV turned out to be correct and achievable, the associated “off-target” immunodeficiency indicates that additional genetic engineering is required to increase anti–PD-1 CAR specificity toward PD-1^+^ CD4 T cells alone and to include safety switches to abrogate anti–PD-1 CAR T cells in vivo to minimize the long-term effects of CAR T cell persistence.

A pertinent question is whether our study allows us to make assumptions about the importance of TFH cells, and whether a CAR T cell approach targeting only one marker of viral persistence is the right strategy is unclear. The short-term clearance of the TFH reservoir prior to the subsequent immunosuppression due to loss of the CD8^+^ memory T cell compartment does not allow us to evaluate what quantitative role TFH depletion plays in long-term persistence. Thus far, a unifying marker for the replication-competent viral reservoir remains elusive. Numerous studies identified PD-1, CTLA4, TIGIT, LAG3, CD44, CD28, CD127, and the IL-21 receptor to be enriched on CD4^+^ T cells that harbor the replication-competent reservoir in blood, gut, and LNs (9–11, 13, 42, 43). TFH cells and tissue-resident memory T cells are important reservoirs during ART in LNs, the gut, and other tissues (9, 42, 44). We recognize other reservoirs of SIV/HIV remain, and what role a second-generation anti–PD-1 CAR T cell will play in affecting such reservoirs remains to be determined. Like ART, it is likely HIV-cure regimens will require a multimodal approach. The ability to utilize CAR T cells with combined ART will help define these issues more clearly in a way that can also be more easily translated to humans.

The development of anti–PD-1 CAR–mediated or -accelerated immunodeficiency characterized by loss of memory CD4^+^ and CD8^+^ T cells, increased SIV replication, and development of an abnormal nodular lymphoma-like condition associated with LCV RNA^+^ cells was likely mediated by one or several mechanisms that hampered the antiviral responses of the T and B cell compartment. With respect to the T cell compartment, PD-1 expression on CD4^+^ and CD8^+^ T cells is quickly upregulated during T cell activation (45) and exhaustion, including HIV-specific T cells (46–48), in the presence of proinflammatory cytokines (49) as well as in homeostatic T cell proliferation during lymphopenia (50). Arguably, all of these mechanisms were present due to CAR T cell infusion, lymphodepletion prior to infusion, and for the SIV-infected RMs, rebound of SIV after cessation of ART may have contributed to the almost complete depletion of the memory T cell compartment during the initial expansion, leading to prolonged lymphopenia. Notably, antibody-mediated depletion of CD8^+^ T cells in SIVmac239-infected RMs showed a similar pattern of an approximately 2-log increase in post-ART plasma viremia relative to controls after cessation of ART (39). We noted stronger depletion of CD8^+^ memory T cells in the SIV-infected RMs compared with the SIV-naive animal, perhaps due to the ongoing viral replication and T cell responses in the SIV-infected RMs. Our data further indicate that anti–PD-1 CAR T cell expansion coincides with a loss of GC Ki67^+^ B cells. One can speculate that the loss of TFH cells, which secrete IL-21 to maintain B cell proliferation in the GC to facilitate the affinity maturation of antibodies (51), could impair de novo antibody response, something that could be investigated in future studies. Whether the loss of GC Ki67^+^ B cells is a consequence of TFH cell depletion or through killing of GC Ki67^+^ B cells by the anti–PD-1 CAR is not known. In contrast, antibody recall responses were not affected in the animals with anti–PD-1 CAR T cell expansion. Ultimately, it is not possible to pinpoint the main factors that contributed to the effects seen in the T and B cell compartment in RMs with CAR T cell expansion; however, the development of anti–PD-1 CAR–related immunodeficiency remains a crucial observation of this study.

While it remains unclear why we did not achieve expansion of the CAR T cells in 3 of our 6 infused animals, the contrast in depletion in the animals with no CAR T cell expansion was useful in defining many of the unexpected observations from our study. The infusate for animal RM6 did not show in vitro killing of KG–PD-1 cells in the potency release assay, which may explain why it did not expand in vivo. However, RM4 and RM2 infusates showed high killing potential, but failed to expand in vivo. Perhaps unappreciated differences in product quality, CAR T cell fratricide, or in vivo expression dynamics of PD-1 may also play a role in inhibiting CAR T cell expansion.

Collectively, these studies indicate both the potential and the need for further development of anti–PD-1 CAR T cells for infectious disease. However, anti–PD-1 CAR T cells require additional safety switches to gain better control over CAR function in vivo as well as additional approaches to increase specificity. As eradication of TFH cells occurred early after infusion, reduction of the long-term immunosuppression by killing/eliminating of anti–PD-1 CAR T cells is one potential approach to achieving benefit from this CAR T cell therapy. To address this, a second-generation anti–PD-1 CAR T cell approach could include a logic-gated synNotch receptor pair in which a primary synNotch receptor targeting one protein controls the gene expression of a CAR targeting a second protein (52, 53). This will decrease systemic CAR function and limit its activity to select tissues. Alternatively, one could use the recently developed logic-gated intracellular network CAR platform (54), which requires binding of 2 CARs simultaneously to induce signaling via LAT and SLP-76. Thus, the anti–PD-1 CAR could be designed to target an additional TFH cell–specific protein such as ICOS besides PD-1 and in turn improve the specificity and safety profile of this approach. Future efforts should therefore aim to integrate increased control over the expression or killing efficacy of the anti–PD-1 CAR within the B cell follicles.

As CAR T cells are increasingly explored in multiple disease indications (55), a second generation anti–PD-1 CAR approach could be considered in oncology to treat selective PD-1–expressing tumors such as angioimmunoblastic T cell lymphoma (56–59) and nodal peripheral T cell lymphomas with TFH markers (60). Another application of anti–PD-1 CAR T cells could be in the treatment of autoimmune diseases. For example, autoreactive T cells (e.g., skin tissue–resident memory T cells in various inflammatory skin diseases or TFH cells in systemic lupus erythematosus [SLE] and rheumatoid arthritis) express PD-1 and are crucial for disease progression in severe autoimmune diseases. Thus, the anti–PD-1 CAR could be used to reset the immune repertoire in a similar manner, as has been recently described with the anti-CD19 CAR to deplete CD19^+^ plasmablasts in refractory SLE (61, 62) and in a patient with antisynthase syndrome (63). While PD-1 is a marker for CD4^+^ T cells enriched with replication-competent provirus, other PD-1 HIV reservoirs such as CTLA4^+^ Tregs (43, 64) have been described.

In summary, these studies describe the development of an anti–PD-1 CAR T cell approach that can efficiently kill PD-1^+^–expressing cells in vitro and in vivo. However, long-term persistence of the anti–PD-1 CAR was associated with CAR-mediated immunodeficiency and lymphopenia, indicating a need for improved CAR design with additional layers of safety to ablate CAR T cell activity in vivo. Also, as the development of ART required a combination of drugs targeting multiple steps of HIV replication, HIV cure will likely require a combinatorial approach as well. Hence, there may be a need to combine the anti–PD-1 CAR with other approaches such as broadly neutralizing antibodies, therapeutic vaccines, or the HIV Env–targeting CAR during ART treatment to fully determine the utility of this approach for eliminating HIV reservoirs.

## Methods

### Sex as a biological variant.

A total of 3 male and 3 female RMs were used for these studies. However, sex as a biological variable was not considered due to the small sample size.

Detailed materials and methods can be found in Supplemental Methods.

### Study approval.

This study was carried out in strict accordance with the standards of the US NIH *Guide for the Care and Use of Laboratory Animals* (National Academies Press, 2011) and was approved by the Institutional Animal Care and Use Committees of the Fred Hutchinson Cancer Research Center and the WaNPRC at the University of Washington and the ONPRC at Oregon Health and Science University. All animals were housed at and included in standard monitoring procedures prescribed by the WaNPRC and ONPRC.

### Data availability.

Values for all data points in graphs are reported in the Supporting Data Values file.

## Author contributions

KE and LC conceived the anti–PD-1 CAR. KE, YF, CWP, BDVM, SD, AAO, and LC designed research. KE, YF, CWP, HP, BDVM, JVS, LJP, AAO, and LC designed the animal experiments. KE, YF, FH, MM, SH, DMD, BDVM, SD, JVS, LJ, JG, and BJR performed research. RDM, MKA, and CSL performed autopsies on SIV-naive and SIV-infected RMs. KE, YF, CWP, SD, LJP, AAO, and LC analyzed and interpreted data, with input from all authors. DHF and HPK provided reagents and helpful discussions. KE, AAO, and LC wrote the manuscript with input from all authors.

## Supplementary Material

Supplemental data

Supporting data values

## Version 1 04/01/2024

Electronic publication
