# Supplementary material for: Anti–PD-1 chimeric antigen receptor T cells efficiently target SIV-infected CD4+ T cells in germinal centers
Source: J Clin Invest. 2024 Apr 1;134(7):e169309. doi: 10.1172/JCI169309 (PMC10977982; doi:10.1172/JCI169309)
Supplement: Supplemental data [file jci-134-169309-s149.pdf]

Supplemental materials

Anti-PD-1 chimeric antigen receptor T cells efficiently target SIV-infected CD4+ T cells in germinal centers.

Karsten Eichholz[1], Yoshinori Fukazawa[2], Christopher W. Peterson[3,4], Francoise Haeseleer[1,4,5], Manuel Medina[2], Shelby Hoffmeister[2], Derick M. Duell[2], Benjamin D. Varco-Merth[2], Sandra Dross[6,7], Haesun Park[2], Caralyn S. Labriola[2], Michael K. Axthelm[2], Robert D. Murnane[6], Jeremy V. Smedley[2], Lei Jin[1], Jiaxin Gong[1], Blake J. Rust[3], Deborah H. Fuller[6,7], Hans-Peter Kiem[1,3,5], Louis J. Picker[2], Afam A. Okoye[2], Lawrence Corey[1,4,5*]

[1]Vaccine and Infectious Disease Division, Fred Hutchinson Cancer Center, Seattle, WA, USA.

[2]Vaccine and Gene Therapy Institute and Oregon National Primate Research Center, Oregon Health & Science University, Beaverton, OR, USA.

[3]Stem Cell and Gene Therapy Program, Fred Hutchinson Cancer Center, Seattle, WA, USA.

[4]Department of Laboratory Medicine, University of Washington, Seattle, WA, USA.

[5]Department of Medicine, University of Washington, Seattle, WA, USA.

[6]Washington National Primate Research Center, Seattle, WA, USA.

[7]Department of Microbiology, University of Washington, Seattle, WA, USA.

***Correspondence**: L. Corey, MD, Fred Hutchinson Cancer Center, 1100 Fairview Ave N, MS E3-300, Seattle, WA 98190 (Lcorey@fredhutch.org).

Methods

Infusion of CAR T cells in SIV-naive and SIV-infected RMs

Infusion of CAR T cells in SIV-naive RM was done in two male RMs (Macaca mulatta), of Indian genetic background. Each recipient received a single dose of cyclophosphamide (Baxter, 30 mg/kg) for lymphodepletion on day -4 and -3 prior to CAR T cell infusion. In addition, anti-IL-6 antibody Tocilizumab was administered at 8 mg/kg once per day for 3 days, starting with the day of CAR T cell infusion in each animal (RM2 and RM1).

Infusion of CAR T cells in SIV-infected RM used four RMs (Macaca mulatta), one male and three females, one Trim5 Q/Q and three Trim5 Q/CypA of Indian genetic background (although1 RM had 28.5% Chinese ancestry). As previously described, these RM were specific pathogen-free; start of ART occurred 12 days post-inoculation with SIVmac239, and each recipient received a single dose of cyclophosphamide (Baxter, 30 mg/kg) for lymphodepletion on day -5 prior to CAR T cell infusion (1–3). In addition, anti-IL-6 antibody Tocilizumab was administered at 8 mg/kg once per day for 3 days, starting with the day of CAR T cell infusion. Anti-PD-1 CAR T cells were infused i.v. at doses of 6×10^6 or 20.8×10^6 EGFRt+ T cells/kg. The CAR T cell dose was decided based on cell availability after production but were in line with previously published CAR T cell experiments in RM that used does in the range of $0.6 - 6.2 \times 10^7$ CAR T cells/kg bodyweight (3–5). ART was stopped on day 14 post-CAR T cell infusion. Whole blood, peripheral lymph nodes (Peri.LN), mesenteric lymph nodes (Mes.LN), spleen, bronchoalveolar lavage (BAL), and bone marrow aspirates (BM), liver, duodenum, and colon were collected longitudinally in all SIV-infected recipient RM as previously described (6–8). Recipient RMs were followed for a minimum of 80 days post-ART cessation for the onset of plasma viremia.

Virus detection assays

Plasma SIV RNA levels were determined using a gag-targeted quantitative real-time/digital RT-PCR format assay, as previously described, with 6 replicate reactions analyzed per extracted sample for assay thresholds of 15 SIV RNA copies/mL (9).

Cell culture

K562 cells (ATCC), Molt-4 cells, clone 8 (NIH HIV Reagent Program) (10), Jurkat and Jurkat Nur77 t2a NeonGreen cells (a generous gift from Dr. Aude Chapuis, Fred Hutchinson Cancer Center, Seattle, WA) were cultured in RPMI1640 (Gibco), 10% fetal bovine serum and antibiotics. Lenti-X 293T cells (Takara) were cultured in DMEM (Gibco), 10% fetal bovine serum and antibiotics.

Anti-PD-1 CAR design and cloning

Six anti-PD-1 CAR constructs based on the anti-PD-1 antibody Pembrolizumab with different VH VL orientation and extracellular linker length were generated in a pcl20 SIV lentivirus transfer vector under the control of a MSCV promoter (11) and cloned between the GM-CSF-signal peptide for membrane targeting and the downstream CD28TM-41BB-CD3z-EGFRt-c46 wPRE expression cassette. In addition, a CXCR5-containing construct as well as binding and signaling-deficient control constructs are based on the VH VL S anti-PD-1 CAR construct.

A pcl20 SIV lentivirus transfer vector containing a MSCV promoter (11) and a GM-CSF-signal peptide CAR-(GM-CSF-signal peptide -binder-extracellular linker-CD28TM-41BB-CD3z)-EGFRt-c46 wPRE expression cassette (3) was amplified by PCR with primers (Primer For 5'- tggaatcagcagaaag -3' and rev 5'-atgttctgggtgctc -3') placed in the GM-CSF peptide and the CD28TM sequence and used as a source to generate a lentiviral backbone to generate the various anti-PD-1 CAR vectors.

The amino acid sequence of heavy and light chain of the anti-PD-1 antibody pembrolizumab was retrieved from the PDB data base (PDB:5DK3) and the paratope regions of the variable heavy (VH) and light (VL) chain was identified with the paratome algorithm (12). We used the first 125 and 134 amino acids of the VH and VL, respectively to design 2 scFv with a VH-VL and VL-VH orientation interconnected with 4 x GGGGS and 15 bp overlaps with the GM-CSF and extracellular linker sequence in silico and reverse translated it in silico. The composition of the extracellular spacer can affect CAR T-cell recognition, (13), thus, the spacer lengths of the CAR were varied by different portions of the IgG4 Fc of various length. Short, medium and long extracellular linker sequences were retrieved from the NCBI database (14) and in silico designed with a 15 base pair overlap with anti-PD-1 scFv and the CD28TM sequence. Putative splice sites in the DNA sequences were identified and removed with the Splice Site Prediction by Neural Network online tool https://www.fruitfly.org/seq_tools/splice.html (15). Gene blocks for the scFv and extracellular linker were ordered from IDT and assembled with the pCL20 lentiviral backbone with a NEBuilder HiFi DNA Assembly Cloning Kit (New England Biolabs) according to the manufacturer's protocol to generate a total of 6 anti-PD-1 CAR with 2 different scFv orientations with each 3 different extracellular linkers.

To co-express CXCR5 for B cell follicle targeting in the same expression cassette as the anti-PD-1 CAR, the CXCR5 cassette was transferred from a pCL20 plasmid containing a CAP256-VRC26.25 CAR EGFRt-c46-CXCR5 wPRE expression cassette (3) by restriction digestion with BspE1 and Not1 and ligation into the pCL20 VH VL S and VH VL M anti-PD-1 CAR EGFRt.

A binding-deficient, a signaling deficient and a binding- and signaling deficient version of the anti-PD-1 CAR in the VH VL S orientation was built based on a pCL20 VH VL S anti-PD-1 CAR EGFRt backbone. The plasmid was sequentially digested with AgeI and BspEI and the original scFv as well as the partial EGFRt domain with or without the CD3ζ domain were amplified by PCR. The binding-deficient VH VL S scFv containing the transmembrane as well as the 41BB domain was ordered from IDT and amino acids implicated in the interaction with PD-1 were changed to either Alanine or Phenylalanine, these are T52A, Y55F, N74A, S76A, N77A, S80A, N81A, R121A, Y123F, R124A, S194A, Y196F, Y215F, Y219F, S257A and D259A.

VH VL S anti-PD-1 CAR:

MLLLVTSLLLCELPHPAFLLIPQVQLVQSGVEVKKPGASVKVSCKASGYTFTNYYMYWVRQAPGQGLEWMGGINPSN
GGTNFNEKFKNRVTLTTDSSTTTAYMELKSLQFDDTAVYYCARRDYRFDMGFDYWGQGTTVTVSSASTKGGGGGSG
GGGSGGGGSEIVLTQSPATLSLSPGERATLSCRASKGVSTSGYSYLHWYQQKPGQAPRLLIYLASYLESGVPARFSGSGS
GTDFTLTISSLEPEDFAVYYCQHSRDLPLTFGGGTKVEIKRTVAAPSVFIFPPSDEQLKSGTAESKYGPPCPPCPMFWVLV
VVGGVLACYSLLVTVAFIIFWVKRGRKKLLYIFKQPFMRPVQTTQEEDGCSCRFPEEEEGGCELRVKFSRSADAPAYQQG
QNQLYNELNLGRREEYDVLDKRRGRDPEMGGKPRRKNPQEGLYNELQKDKMAEAYSEIGMKGERRRGKGHDGLYQ
GLSTATKDTYDALHMQALPPR

VH VL M anti-PD-1 CAR:

MLLLVTSLLLCELPHPAFLLIPQVQLVQSGVEVKKPGASVKVSCKASGYTFTNYYMYWVRQAPGQGLEWMGGINPSN
GGTNFNEKFKNRVTLTTDSSTTTAYMELKSLQFDDTAVYYCARRDYRFDMGFDYWGQGTTVTVSSASTKGGGGGSG
GGGSGGGGSEIVLTQSPATLSLSPGERATLSCRASKGVSTSGYSYLHWYQQKPGQAPRLLIYLASYLESGVPARFSGSGS
GTDFTLTISSLEPEDFAVYYCQHSRDLPLTFGGGTKVEIKRTVAAPSVFIFPPSDEQLKSGTAESKYGPPCPPCPGQPREPQ
VYTLPPSQEEMTKNQVSLTCLVKGFYPSDIAVEWESNGQPENNYKTTPPVLDSDGSFFLYSRLTVDKSRWQEGNVFSCS
VMHEALHNHYTQKSLSLSLGKMFWVLVVVGGVLACYSLLVTVAFIIFWVKRGRKKLLYIFKQPFMRPVQTTQEEDGCSC
RFPEEEEGGCELRVKFSRSADAPAYQQGQNQLYNELNLGRREEYDVLDKRRGRDPEMGGKPRRKNPQEGLYNELQKD
KMAEAYSEIGMKGERRRGKGHDGLYQGLSTATKDTYDALHMQALPPR

VH VL L anti-PD-1 CAR:

MLLLVTSLLLCELPHPAFLLIPQVQLVQSGVEVKKPGASVKVSCKASGYTFTNYYMYWVRQAPGQGLEWMGGINPSN
GGTNFNEKFKNRVTLTTDSSTTTAYMELKSLQFDDTAVYYCARRDYRFDMGFDYWGQGTTVTVSSASTKGGGGGSG
GGGSGGGGSEIVLTQSPATLSLSPGERATLSCRASKGVSTSGYSYLHWYQQKPGQAPRLLIYLASYLESGVPARFSGSGS
GTDFTLTISSLEPEDFAVYYCQHSRDLPLTFGGGTKVEIKRTVAAPSVFIFPPSDEQLKSGTAESKYGPPCPPCPGPSVFLFP

PKPKDTLMISRTPEVTCVVVDVSQEDPEVQFNWYVDGVEVHNAKTKPREEQFQSTYRVVSVLTVLHQDWLNGKEYKC
KVSNKGLPSSIEKTISKAKGQPREPQVYTLPPSQEEMTKNQVSLTCLVKGFYPSDIAVEWESNGQPENNYKTTPPVLDSD
GSFFLYSRLTVDKSRWQEGNVFSCSVMHEALHNHYTQKSLSLSLGKMFWVLVVVGGVLACYSLLVTVAFIIFWVKRGR
KKLLYIFKQPFMRPVQTTQEEDGCSCRFPEEEEGGCELRVKFSRSADAPAYQQGQNQLYNELNLGRREEYDVLDKRRG
RDPEMGGKPRRKNPQEGLYNELQKDKMAEAYSEIGMKGERRRGKGHDGLYQGLSTATKDTYDALHMQALPPR

VL VH S anti-PD-1 CAR:

MLLLVTSLLLCELPHPAFLLIPEIVLTQSPATLSLSPGERATLSCRASKGVSTSGYSYLHWYQQKPGQAPRLLIYLASYLESG
VPARFSGSGSGTDFTLTISSLEPEDFAVYYCQHSRDLPLTFGGGTKVEIKRTVAAPSVFIFPPSDEQLKSGTAGGGGSGGG
GSGGGGSQVQLVQSGVEVKKPGASVKVSCKASGYTFTNYYMYWVRQAPGQGLEWMGGINPSNGGTNFNEKFKNR
VTLTTDSSTTTAYMELKSLQFDDTAVYYCARRDYRFDMGFDYWGQGTTVTVSSASTKGESKYGPPCPPCPMFWVLVV
VGGVLACYSLLVTVAFIIFWVKRGRKKLLYIFKQPFMRPVQTTQEEDGCSCRFPEEEEGGCELRVKFSRSADAPAYQQG
QNQLYNELNLGRREEYDVLDKRRGRDPEMGGKPRRKNPQEGLYNELQKDKMAEAYSEIGMKGERRRGKGHDGLYQ
GLSTATKDTYDALHMQALPPR

VL VH M anti-PD-1 CAR:

MLLLVTSLLLCELPHPAFLLIPEIVLTQSPATLSLSPGERATLSCRASKGVSTSGYSYLHWYQQKPGQAPRLLIYLASYLESG
VPARFSGSGSGTDFTLTISSLEPEDFAVYYCQHSRDLPLTFGGGTKVEIKRTVAAPSVFIFPPSDEQLKSGTAGGGGSGGG
GSGGGGSQVQLVQSGVEVKKPGASVKVSCKASGYTFTNYYMYWVRQAPGQGLEWMGGINPSNGGTNFNEKFKNR
VTLTTDSSTTTAYMELKSLQFDDTAVYYCARRDYRFDMGFDYWGQGTTVTVSSASTKGESKYGPPCPPCPGQPREPQV
YTLPPSQEEMTKNQVSLTCLVKGFYPSDIAVEWESNGQPENNYKTTPPVLDSDGSFFLYSRLTVDKSRWQEGNVFSCSV
MHEALHNHYTQKSLSLSLGKMFWVLVVVGGVLACYSLLVTVAFIIFWVKRGRKKLLYIFKQPFMRPVQTTQEEDGCSCR
FPEEEEGGCELRVKFSRSADAPAYQQGQNQLYNELNLGRREEYDVLDKRRGRDPEMGGKPRRKNPQEGLYNELQKDK
MAEAYSEIGMKGERRRGKGHDGLYQGLSTATKDTYDALHMQALPPR

VL VH L anti-PD-1 CAR:

MLLLVTSLLLCELPHPAFLLIPEIVLTQSPATLSLSPGERATLSCRASKGVSTSGYSYLHWYQQKPGQAPRLLIYLASYLESG
VPARFSGSGSGTDFTLTISSLEPEDFAVYYCQHSRDLPLTFGGGTKVEIKRTVAAPSVFIFPPSDEQLKSGTAGGGGSGGG
GSGGGGSQVQLVQSGVEVKKPGASVKVSCKASGYTFTNYYMYWVRQAPGQGLEWMGGINPSNGGTNFNEKFKNR
VTLTTDSSTTTAYMELKSLQFDDTAVYYCARRDYRFDMGFDYWGQGTTVTVSSASTKGESKYGPPCPPCPGPSVFLFPP
KPKDTLMISRTPEVTCVVVDVSQEDPEVQFNWYVDGVEVHNAKTKPREEQFQSTYRVVSVLTVLHQDWLNGKEYKCK
VSNKGLPSSIEKTISKAKGQPREPQVYTLPPSQEEMTKNQVSLTCLVKGFYPSDIAVEWESNGQPENNYKTTPPVLDSDG
SFFLYSRLTVDKSRWQEGNVFSCSVMHEALHNHYTQKSLSLSLGKMFWVLVVVGGVLACYSLLVTVAFIIFWVKRGRKK

LLYIFKQPFMRPVQTTQEEDGCSCRFPEEEEGGCELRVKFSRSADAPAYQQGQNQLYNELNLGRREEYDVLDKRRGRD PEMGGKPRRKNPQEGLYNELQKDKMAEAYSEIGMKGERRRGKGHDGLYQGLSTATKDTYDALHMQALPPR

VH VL S anti-PD-1 CAR BD (binding-defective):

MLLLVTSLLLCELPHPAFLLIPQVQLVQSGVEVKKPGASVKVSCKASGYTFANYFMYWVRQAPGQGLEWMGGIAPAA GGAAFNEKFKNRVTLTTDSSTTTAYMELKSLQFDDTAVYYCARADFAFDMGFDYWGQGTTVTVSSASTKGGGGGSG GGGSGGGGSEIVLTQSPATLSLSPGERATLSCRASKGVSTAGFSYLHWYQQKPGQAPRLLIFLASFLESGVPARFSGSGS GTDFTLTISSLEPEDFAVYYCQHARALPLTFGGGTKVEIKRTVAAPSVFIFPPSDEQLKSGTAESKYGPPCPPCPMFWVLV VVGGVLACYSLLVTVAFIIFWVKRGRKKLLYIFKQPFMRPVQTTQEEDGCSCRFPEEEEGGCELRVKFSRSADAPAYQQG QNQLYNELNLGRREEYDVLDKRRGRDPEMGGKPRRKNPQEGLYNELQKDKMAEAYSEIGMKGERRRGKGHDGLYQ GLSTATKDTYDALHMQALPPR

VH VL S anti-PD-1 CAR ΔCD3z:

MLLLVTSLLLCELPHPAFLLIPQVQLVQSGVEVKKPGASVKVSCKASGYTFTNYYMYWVRQAPGQGLEWMGGINPSN GGTNFNEKFKNRVTLTTDSSTTTAYMELKSLQFDDTAVYYCARRDYRFDMGFDYWGQGTTVTVSSASTKGGGGGSG GGGSGGGGSEIVLTQSPATLSLSPGERATLSCRASKGVSTSGYSYLHWYQQKPGQAPRLLIYLASYLESGVPARFSGSGS GTDFTLTISSLEPEDFAVYYCQHSRDLPLTFGGGTKVEIKRTVAAPSVFIFPPSDEQLKSGTAESKYGPPCPPCPMFWVLV VVGGVLACYSLLVTVAFIIFWVKRGRKKLLYIFKQPFMRPVQTTQEEDGCSCRFPEEEEGGCEL

VH VL S anti-PD-1 CAR BD ΔCD3z

MLLLVTSLLLCELPHPAFLLIPQVQLVQSGVEVKKPGASVKVSCKASGYTFANYFMYWVRQAPGQGLEWMGGIAPAA GGAAFNEKFKNRVTLTTDSSTTTAYMELKSLQFDDTAVYYCARADFAFDMGFDYWGQGTTVTVSSASTKGGGGGSG GGGSGGGGSEIVLTQSPATLSLSPGERATLSCRASKGVSTAGFSYLHWYQQKPGQAPRLLIFLASFLESGVPARFSGSGS GTDFTLTISSLEPEDFAVYYCQHARALPLTFGGGTKVEIKRTVAAPSVFIFPPSDEQLKSGTAESKYGPPCPPCPMFWVLV VVGGVLACYSLLVTVAFIIFWVKRGRKKLLYIFKQPFMRPVQTTQEEDGCSCRFPEEEEGGCEL

Generation of K562 cells PD-1-GFP and KG PD-1 cells

PD-1 expressing target cells were generated by lentiviral transduction with a PD-1 expressing HIV-based lentivirus followed by fluorescence assisted cell sorting for PD-1 expression and absolute quantification of PD-1 molecules per cell. HIV-based lentivirus transfer vector carrying a PD-1 GFPSpark expression cassette under the control of a CMV promoter was purchased from Sino Biological (HG10377-ACGLN). GFP Spark was removed by PCR of the PD-1 nucleotide sequence, which was then reinserted into the parental vector between the NheI and XhoI site using the NEBuilder HiFi DNA Assembly kit (New England Biolabs), and the

plasmid was renamed pLV PD-1. Lentivirus was produced using the psPax2 and pMD2.G plasmid on 293T cells. K562 cells were transduced to express PD-1-GFP and sorted to obtain three cell lines (K562 PD-1-GFP high, K562 PD-1-GFP medium, K562 PD-1-GFP low) that express differential levels of PD-1 and GFP. K562 GFP cells and K562 expressing GFP and PD-1 (KG PD-1 cells) from two separate expression cassettes were made by sequential transduction with CL20 MSCV GFP lentivirus, fluorescence assisted cell sorting for GFP expression and a second transduction with LV PD-1 and fluorescence assisted cell sorting for PD-1 expression. Absolute quantification of PD-1 cell surface expression of K562 PD-1-GFP high, K562 PD-1-GFP medium, K562 PD-1-GFP low, K562 GFP control cells was done by flow cytometry with PE-conjugated anti-PD-1 antibody (clone EH12.2H7, biolegend) and the Quantibrite kit (Becton Dickinson Biosciences). Cell surface expression was calculated at fluorescence saturation.

Lentivirus production

Small scale batches of SIV-based lentiviruses were produced in Lenti-X 293T cells (Takara Bio). Cells were cultured with Dulbecco's modified Eagle's medium containing 10% fetal bovine serum (FBS) and 100 U/mL Pen/Strep were transfected using polyethylimine (linear, MW 25000, Polysciences) with four plasmids, 10 µg of the CAR transfer vector, 6 µg of the pCAG-SIVgprre plasmid carrying the gag/pol and rev responsive element, 2 µg of the rev/tat expression plasmid pCAG4-RTR-SIV, and 2 µg of the pMD2.CocalG containing the glycoprotein G of the cocal virus (a generous gift from Dr. Kiem, Fred Hutchinson Cancer Research Center, Seattle, WA). Sixteen hours after transfection, the medium was replaced. The lentivirus-containing media was harvested 30 h later and cleared by centrifugation at 500 x g for 5 min followed by filtration on a 0.45 µm filter (Millipore-Sigma). The lentivirus preparation was then layered on top of a 10% sucrose, 0.5 mM ethylene diamine tetra acetic acid (EDTA) in PBS (Gibco) and concentrated by centrifugation at 3,000 g overnight at 4°C. The supernatant was discarded, and the lentivirus pellet was resuspended in PBS (100 x concentration), and then stored at -80°C.

The lentivirus preparation was titrated by adding various amounts of lentivirus to Jurkat cells on fibronectin-coated plates followed by spinoculation for 2 h at 1,200 x g. Plates were coated with 20µg/mL fibronectin (Millipore-Sigma) in PBS overnight at 4°C. Two days later, EGFR expression was assessed by flow cytometry.

T cell culture and CAR T cell expansion

CD8+ and CD4+ rhesus macaque T cells were isolated from frozen PBMC by sequential positive selection of CD8 T cells with nonhuman primate CD8 microbeads (Miltenyi) followed by enrichment of CD4 T cells

with an EasySep Nonhuman primate CD4+ T cell isolation kit (Stemcell Technologies). T cell activation and culture was performed as previously described in X-VIVO15, 10% FBS (Gibco), 50 µM β-mercaptoethanol (SigmaAldrich), antibiotics (Gibco), (coined cX15) and cytokines (3, 16).

Production of CAR T cell infusion products

Infusion products were prepared as previously described for 7-10 days in vitro (3, 5). Before infusion, a small fraction of the CAR T-cell product was reserved for flow cytometry.

CAR cell surface expression and paratope accessibility

CAR cell surface expression was assessed with a PE-or biotin-conjugated human PD-1 Fc fusion protein (PD-1-Fc-PE) (Acrobiosystems) on Jurkat or Molt-4 cells that expressed the various anti-PD-1 CAR-EGFRt. The biotin-conjugated PD-1-Fc protein was counterstained with BV421-conjugated Streptavidin (Invitrogen). In brief, 1 x 10^5 cells were stained with PD-1-Fc-PE and mouse anti-EGFRt antibody clone 31G7 Alexa647 (NBP2-47740AF647/NovusBio) or PD-1-Fc-biotin Streptavidin BV421 in combination with either PE-conjugated Erbitux or anti-EGFRt antibody clone Emab134 (566778,Becton-Dickinson) and cell surface expression was assessed by flow cytometry.

Nur77 assay for tonic signaling and for antigen-specific signaling

2 x 10^5 Jurkat cells that expressed the various CAR-EGFRt expression cassettes were plated in cRPMI in a 96 well plate. The cells were stimulated with 2.5 µL anti-CD3/anti-28 immunocult activator (Stemcell) in cRPMI, 2 x 10^5 K562 PD-1-GFP, 2 x 10^5 GFP or vehicle for 4 h. EGFRt cell surface expression was expressed with mouse anti-EGFRt antibody clone 31G7 Alexa647 NBP2-47740AF647/NovusBio). Subsequently, the cells were stained with the Transcription Factor Buffer Set (Becton-Dickinson) and mouse-Nur77 antibody clone 12.14 PE (12-5965-82/eBioscience). Antigen-independent (tonic signaling) and antigen-dependent activation were assessed by flow cytometry.

CAR T cell killing live cell imaging assay

In a BioCoat 96-Well, Poly-D Lysine-Treated, Flat-Bottom 96 well plate (Corning), 2 x 10^4 target cells (K562 PD-1 GFP high, medium, low, KG PD-1 cells or autologous primary SIVmac239 NefIRESGFP-infected CD4 T cells) in 100 µL cX15 were combined with 100 µL cX15 containing 6 x 10^4, 2 x 10^4 or 0.66 x 10^4 effector cells (anti-PD-1 CAR or control T cells as indicated) to reach effector to target ratios of 3:1; 1:1 or 1:3. E:T ratios were calculated based on the %EGFRt+ cells. Each condition was run in triplicate. GFP expression

was monitored, and 5 images per well were acquired every 3 h with an Incucyte S3 Live cell imaging system placed in standard cell culture incubator at 37*C, 5% CO_2 for up to 4 days.

CAR T cell detection and immunophenotyping

To evaluate the CAR T cells *ex vivo*, whole blood and mononuclear cell preparations from tissue biopsies were stained with antibodies labeled with fluorochromes for cytometric analysis.

All samples from the study in SIV-naive RM were viability stained with live dead aqua (L34957/LifeTechnologies) at room temperature, anti-CCR7 (568681/3D12: BUV395, BD Biosciences) and anti-CXCR5 (25-9185-42/MU5UBEE: PE-Cy7, eBioscience) at 37°C, and anti-CD28 (741168/CD28.2: BUV496, BD Biosciences), anti-CD137 (561702/4B4-1: APC, BD Biosciences), anti-CD8 (557760/RPA-T8: APC-Cy7, BD Biosciences), anti-TIGIT (11-9500-42/MBSAF43: FITC, ThermoFisher) anti-CD95 (562648/DX2: BV421, BD Biosciences), anti-CD20 (302332/2H7: BV570, BioLegend), anti-CD4 (317438/OKT4: BV605, BioLegend), anti-CD3 (563916/Sp34-2: BV650, BD Biosciences), anti-HLA-DR (307644/L243: BV711, BioLegend), anti-PD-1 (329930/EH12.2H7: BV786, BioLegend), anti-CD45 (562394/D058-1283: PECF594, BD Biosciences), and anti-EGFR (PE-conjugated Cetuximab, kindly provided by Juno Therapeutics, now part of Bristol Myers Squibb) at 4°C. Cells were then washed and permeabilized for ICS with BD Cytofix/Cytoperm fixation/ permeabilization solution kit as per kit instructions and stained with anti-Ki67 (561277/B56: AF700, BD Biosciences) in a subset of experiments. Individual FMO controls for EGFR staining were run alongside full stained cells in all experiments. Experiments were acquired on a 5 laser LSRII 18 color analyzer (BD Bioscience) and analyzed using FlowJo v10 (BD).

All samples from the study in SIV-infected RM (100 µL whole blood or 10^6 small lymphocytes from tissue samples or cultured CAR T cells) were initially stained with anti-EGFR (Hu1: Biotin, R&D Systems, FAB9577B-100), and Live/Dead Fixable Aqua Dead Cell Stain Kit (Thermo Fisher, L34957) for 30 min. After washing, the samples were stained with surface antibodies 30 min: anti-CD45 (D058-1283: BUV395, BD Biosciences, 564099), anti-CD8a (SK1: BUV737, BD Biosciences, Custom Bulk 624235), streptavidin (BV421, BD Biosciences, Custom Bulk 624337), anti-CXCR5 (MU5UBEE: Super Bright 600, eBioscience, 63-9185-42), anti-CCR7 (150503: BV711, BD Biosciences, Custom Bulk 624386), anti-CD4 (L200: BV786, BD Biosciences, Custom Bulk 624159), anti-CD95 (DX2: PE, BioLegend, Custom Bulk 94203), anti-CD28 (CD28.2: PE-DAZZ, BioLegend, Custom Bulk 93364), anti-PD-1 (J105, PerCP-eFluor710, eBioscience Custom Bulk CUST00656), anti-CCR5 (3A9: APC, BD Biosciences, Custom Bulk 624076), anti-CD3 (SP34-2, Alexa Fluor700, BD Biosciences, 557917), and anti-CD20 (2H7: APC/Fire750, BioLegend,

Custom Bulk 93924). Intracellular staining with anti-Ki67 (B56: FITC, BD Biosciences, Custom Bulk 624046) was performed for 45 min after lyse/ Fix (BD Biosciences) and permeabilizations.

Polychromatic (8–14 parameter) flow-cytometric analysis was performed on a LSR II BD instrument as previously described (9). List mode multiparameter data files were analyzed using FlowJo v10 (BD).

Viral neutralization assays

Neutralization was evaluated as previously described (17).

Microscopy

Tissue samples were collected and combined immunofluorescence and CAR RNA FISH was performed as previously described with some modifications (18). After sectioning, formalin-fixed, paraffin-embedded (FFPE) tissue section were heated at 60ºC for 1 h, dewaxed and antigen retrieval was done on a Leica Bond RX with Leica Bond Epitope Retrieval Solution 2 (ACDbio) for 15 min at 95ºC (18). Subsequently, lymphoid tissue sections were digested for 15 min with protease III diluted 1 to 5 with PBS at 40°C. WPRE-01 probes (450261-C1, ACDbio) directed against the Woodchuck Hepatitis Virus Posttranscriptional Regulatory Element, located within the 3'UTR of the CAR expression cassette, were used to detect CAR T cells. Probes against mmu CD8α (481881-C3, ACDbio) and SIVmac239 (312011-C2 ACDbio) (19) were used as indicated. Fluorescence signal was developed with multiplex RNAscope version 2 kit (ACDbio) using Opal540, Opal620 or Opal650 (PerkinElmer). To perform immunofluorescence after developing the last RNA FISH signal, tissue sections were washed once with PBS and blocked with 2% donkey serum (Jackson ImmunoResearch), 1x Casein (Vector Laboratories), 2% BSA (Gemini Bio-Products) and 0.2% v/v Triton X-100 (SigmaAldrich) in dH_2O for 30 min. The tissue sections were incubated with the primary antibodies monoclonal rat-anti CD3ε (1:200, clone CD3-12, Abcam), monoclonal mouse ant-CD20 (1:50, clone L26, ebiosciences), and polyclonal rabbit anti-PD-1 (1:50, HPA035981, Sigma Aldrich), diluted in blocking buffer overnight at 4°C. Subsequently, the sections were washed 3 times briefly with PBS Tween (0.05% v/v) and incubated with the secondary antibodies donkey anti-rat Alexa488 (1:100, Invitrogen) donkey anti-rabbit AlexaFlour546 (1:100, Invitrogen) and donkey anti-mouse DyLight680 (1:100, Invitrogen) diluted in blocking buffer. The sections were washed with PBS Tween (0.05% v/v), counterstained with DAPI (ACDbio) and mounted with ProLong Gold antifade (Thermofisher).

Images were acquired with a Leica SP8 confocal microscope equipped with laser lines at 405 nm, 440 nm, and adjustable white light laser using a 20x or 40x objective.

Statistics

Statistics were analyzed using a 2-way ANOVA with Tukey's multiple comparisons test at each time point. P values are reported as *=p<0.05, **= p<0.01, ***= p<0.001, **** = p<0.0001. GraphPad Prism 9 (GraphPad Software) was used for statistical analysis.

Study approval

This study was carried out in strict accordance with the standards of the US National Institutes of Health Guide for the Care and Use of Laboratory Animals, and was approved by the Institutional Animal Care and Use Committees of the Fred Hutchinson Cancer Research Center and Washington National Primate Research Center (WaNPRC) at University of Washington, and the Oregon National Primate Research Center (ONPRC) at Oregon Health and Science University. All animals were housed at and included in standard monitoring procedures prescribed by the WaNPRC and ONPRC.

Data availability

The data sets presented in the current study can be accessed in the "supporting data values" XLS file and are also available from the corresponding author upon reasonable request.

Supplemental references

1. Fennessey CM et al. Genetically-barcoded SIV facilitates enumeration of rebound variants and estimation of reactivation rates in nonhuman primates following interruption of suppressive antiretroviral therapy. *PLoS Pathog.* 2017;13(5):e1006359.

2. Prete GQ Del et al. Short communication: comparative evaluation of coformulated injectable combination antiretroviral therapy regimens in simian immunodeficiency virus-infected rhesus macaques. *AIDS Res. Hum. Retroviruses* 2016;32(2):163–168.

3. Haeseleer F et al. Immune inactivation of anti-simian immunodeficiency virus chimeric antigen receptor T cells in rhesus macaques. *Mol. Ther. Methods Clin. Dev.* 2021;22:304–319.

4. Taraseviciute A et al. Chimeric antigen receptor T cell–mediated neurotoxicity in nonhuman primates. *Cancer Discov.* 2018;8(6):750–763.

5. Rust BJ et al. Robust expansion of HIV CAR T cells following antigen boosting in ART-suppressed nonhuman primates. *Blood* 2020;136(15):1722–1734.

6. Smedley J et al. Laparoscopic Technique for Serial Collection of Para-Colonic, Left Colic, and Inferior Mesenteric Lymph Nodes in Macaques. *PLoS One* 2016;11(6):e0157535.

7. Zevin AS et al. Laparoscopic technique for serial collection of liver and mesenteric lymph nodes in macaques. *J. Vis. Exp.* 2017;(123):55617.

8. Id CM et al. Antimicrobial prophylaxis does not improve post-surgical outcomes in SIV/SHIV-uninfected or SIV/SHIV-infected macaques (Macaca mulatta and Macaca fascicularis) based on a retrospective analysis. *PLoS One* 2022;17(4):e0266616.

9. Hansen SG et al. Immune clearance of highly pathogenic SIV infection. *Nature* 2013;502(7469):100–104.

10. Kikukawa R et al. Differential susceptibility to the acquired immunodeficiency syndrome retrovirus in cloned cells of human leukemic T-cell line molt-4. *JVI* 1986;57(3):1159–1162.

11. Hanawa H et al. Efficient gene transfer into rhesus repopulating hematopoietic stem cells using a simian immunodeficiency virus-based lentiviral vector system. *Blood* 2004;103(11):4062–4069.

12. Kunik V, Ashkenazi S, Ofran Y. Paratome: An online tool for systematic identification of antigen-binding regions in antibodies based on sequence or structure. *Nucleic Acids Res.* 2012;40(W1):W521–W524.

13. Hudecek M et al. The Nonsignaling Extracellular Spacer Domain of Chimeric Antigen Receptors Is Decisive for In Vivo Antitumor Activity. *Cancer Immunol. Res.* 2015;3(2):125–135.

14. Zah E, Lin M-Y, Silva-Benedict A, Jensen MC, Chen YY. T Cells Expressing CD19/CD20 Bispecific

Chimeric Antigen Receptors Prevent Antigen Escape by Malignant B Cells. *Cancer Immunol. Res.* 2016;4(6):498–508.

15. Reese MG, Eeckman FH, Kulp D, Haussler D. Improved Splice Site Detection in Genie. *J. Comput. Biol.* 1997;4(3):311–323.

16. Haeseleer F et al. Real-time killing assays to assess the potency of a new anti-simian immunodeficiency virus chimeric antigen receptor T cell. *AIDS Res. Hum. Retroviruses* 2020;36(12):998–1009.

17. Roederer M et al. Immunological and virological mechanisms of vaccine-mediated protection against SIV and HIV. *Nature* 2014;505(7484):502–508.

18. Eichholz K et al. A CAR RNA FISH assay to study functional and spatial heterogeneity of chimeric antigen receptor T cells in tissue. *Sci. Rep.* 2021;11:12921.

19. Deleage C et al. Defining HIV and SIV Reservoirs in Lymphoid Tissues. *Pathog. Immun.* 2016;1(1):68–106.

20. Horita S et al. High-resolution crystal structure of the therapeutic antibody pembrolizumab bound to the human PD-1. *Sci. Rep.* 2016;6:35297.

21. Madeira F et al. The EMBL-EBI search and sequence analysis tools APIs in 2019. *Nucleic Acids Res.* 2019;47(W1):W636–W641.

Supplemental Figures and Tables

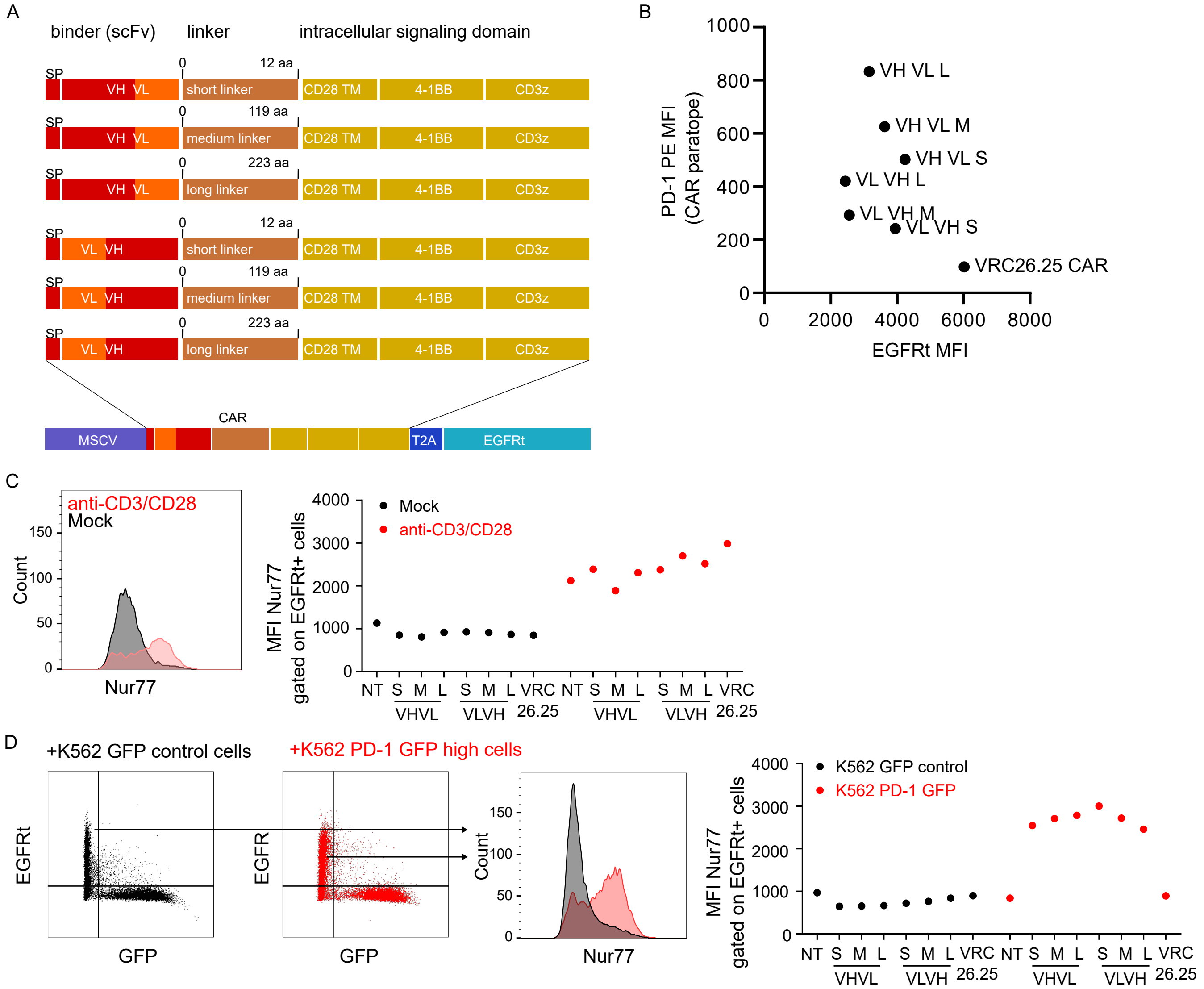

Supplemental Figure 1: Anti-PD-1 CARs signal specifically in presence of PD-1+ cells with low tonic signaling.

A diagram illustrating six anti-PD-1 CARs that differ in the VH VL orientation of the pembrolizumab-derived scFv and the linker length between the transmembrane domain and scFv. These CARs were cloned into an SIV-based lentiviral vector under the control of an MSCV promoter. The CAR expression cassette contains also a CD28 TM, 4-1BB intracellular domain and CD3z domain upstream of a truncated EGFRt for marking. CAR and EGFRt sequences were interspersed with a t2a self-cleaving peptide (A). The six anti-PD-1 CAR vary in their paratope accessibility assessed with PD-1 Fc in Jurkat cells expressing the anti-PD-1 CAR or a VRC26.25-based CAR (data from one representative experiment are shown, n=3) (B). Jurkat cells expressing the six anti-PD-1 CAR constructs exhibit low tonic signaling at baseline comparable to a control VRC26.25 CAR and upregulate Nur77 expression after induction with an anti-CD3/CD28 reagent for 4 h (data from one representative experiment are shown, n=2) (C). Jurkat cells expressing the six anti-PD-1 CAR constructs upregulate Nur77 expression when cocultured for 4 h with K562 PD-1 GFP cells but not K562 GFP control cells. The control VRC26.25 CAR did not upregulate Nur77 in presence of PD-1-expressing cells (data from one representative experiment are shown, n=2) (D).

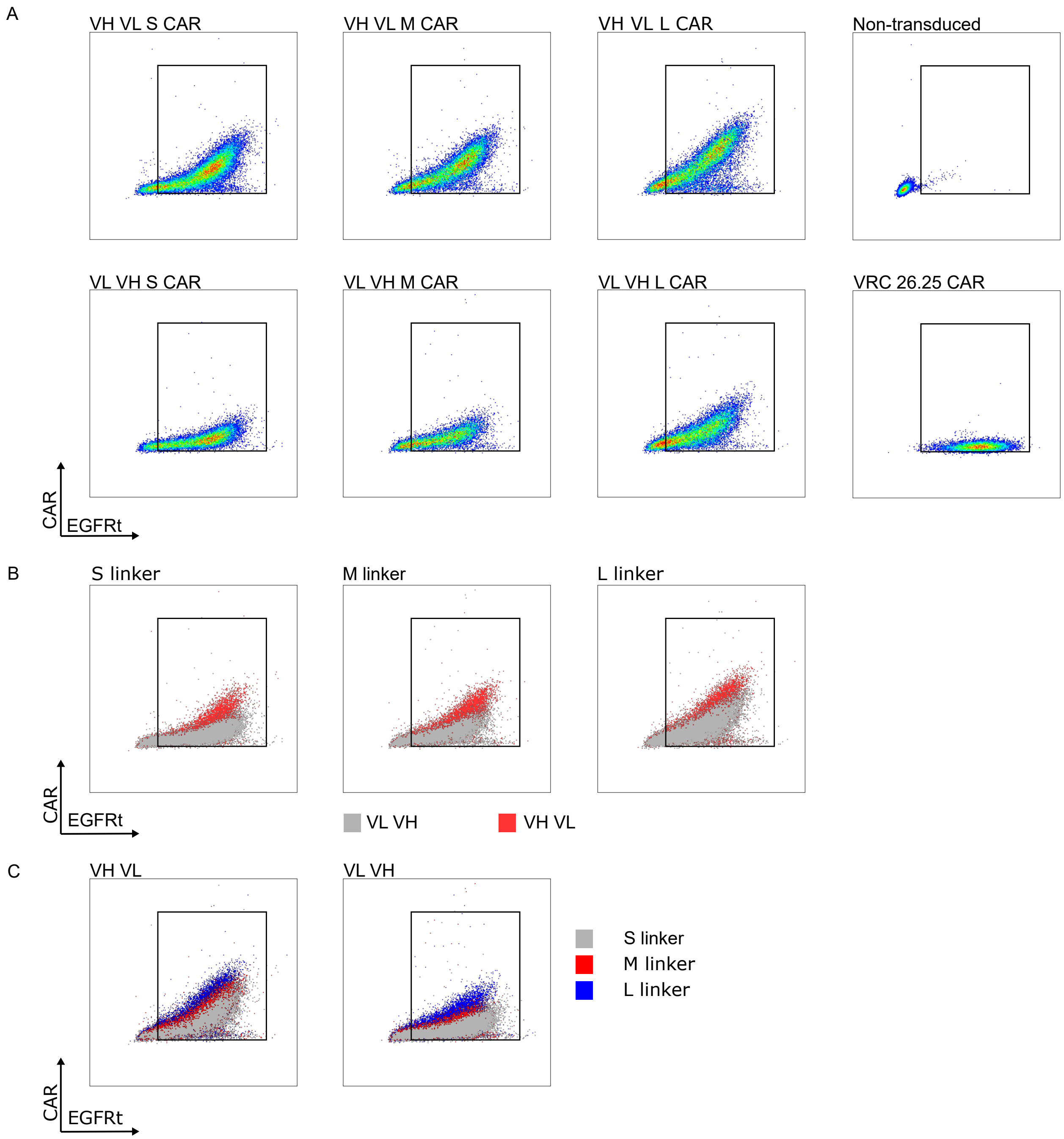

Supplemental Figure 2: VH VL orientation and linker lengths affects paratope accessibility. Flow cytometry plots showing expression of EGFRt and paratope accessibility measured with PE-conjugated PD-1 Fc chimera of non-transduced Jurkat cells, a VRC26.25 CAR and the six anti-PD-1 CAR(A). Comparison of paratope accessibility of the VH VL and VL VH scFv of anti-PD-1 CAR having the same linker length (B). Comparison of paratope accessibility dependent on the linker length for the VH VL and VL VH scFv orientation, respectively (C). (data from one representative experiment are shown, n=3).

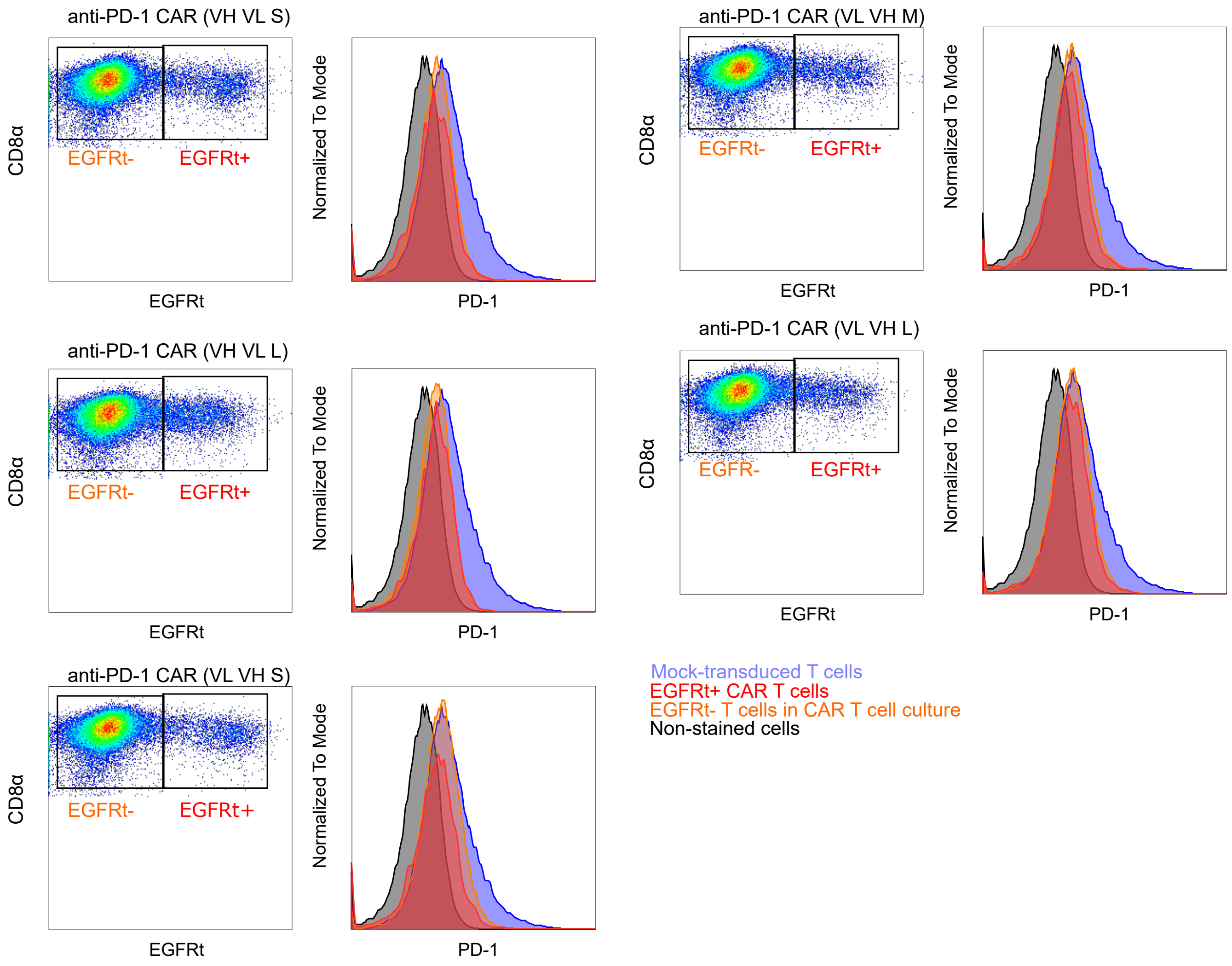

Supplemental Figure 3: PD-1 expression on primary anti-PD-1 CAR T cells.
RM CD8+ T cells transduced with the five remaining anti-PD-1 CAR constructs (VH VL M, VH VL L, VL VH S, VL VH M, VL VH L) and analyzed by flow cytometry detect EGFRt expression as a surrogate marker for CAR transduction. Gating on EGFRt+ and EGFRt- cells in the culture revealed a loss of PD-1 expression on CAR T and bystander cells in CAR T cell culture in comparison to non-transduced T cells (data from one representative experiment are shown, n=2).

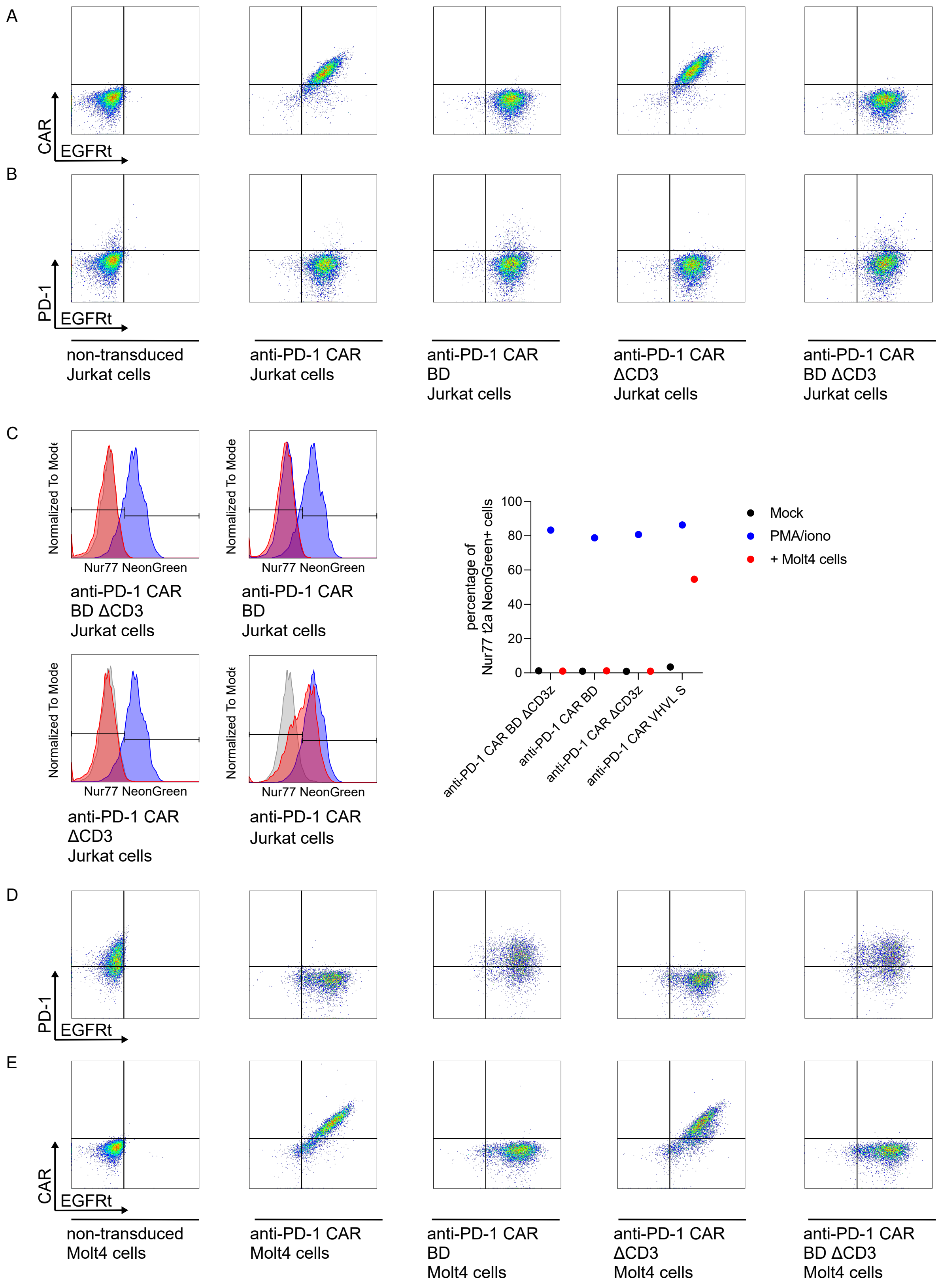

Supplemental Figure 4: Interaction of the anti-PD-1 CAR scFv and PD-1 in cis.
Expression of EGFRt, CAR paratope (data from one representative experiment are shown, n=3) (A) and PD-1 (data from one representative experiment are shown, n=3) (B) on Jurkat cells containing a

t2a -NeonGreen reporter downstream of the Nur77 gene. The cells were transduced with anti-PD-1 CAR (VHVLS), and binding- or signaling deficient versions of the same CAR (anti-PD-1 CAR BD, anti-PD-1 CAR ΔCD3ζ, and anti-PD-1 CAR BD ΔCD3ζ). Induction of Nur77 t2a NeonGreen expression in anti-PD-1 CAR, anti-PD-1 CAR BD, anti-PD-1 CAR ΔCD3ζ, and anti-PD-1 CAR BD ΔCD3ζ Jurkat reporter cell lines in presence of PD-1-expressing Molt 4 cells. PMA/ionomycin was used as a positive control (data from one representative experiment are shown, n=3) (C). Expression of EGFRt, PD-1 (data from one representative experiment are shown, n=3) (D) and CAR paratope (E) on Molt4 cells, which were transduced with anti-PD-1 CAR (VHVLS), and binding- or signaling deficient versions of the same CAR (anti-PD-1 CAR BD, anti-PD-1 CAR ΔCD3ζ, and anti-PD-1 CAR BD ΔCD3ζ) (data from one representative experiment are shown, n=3).

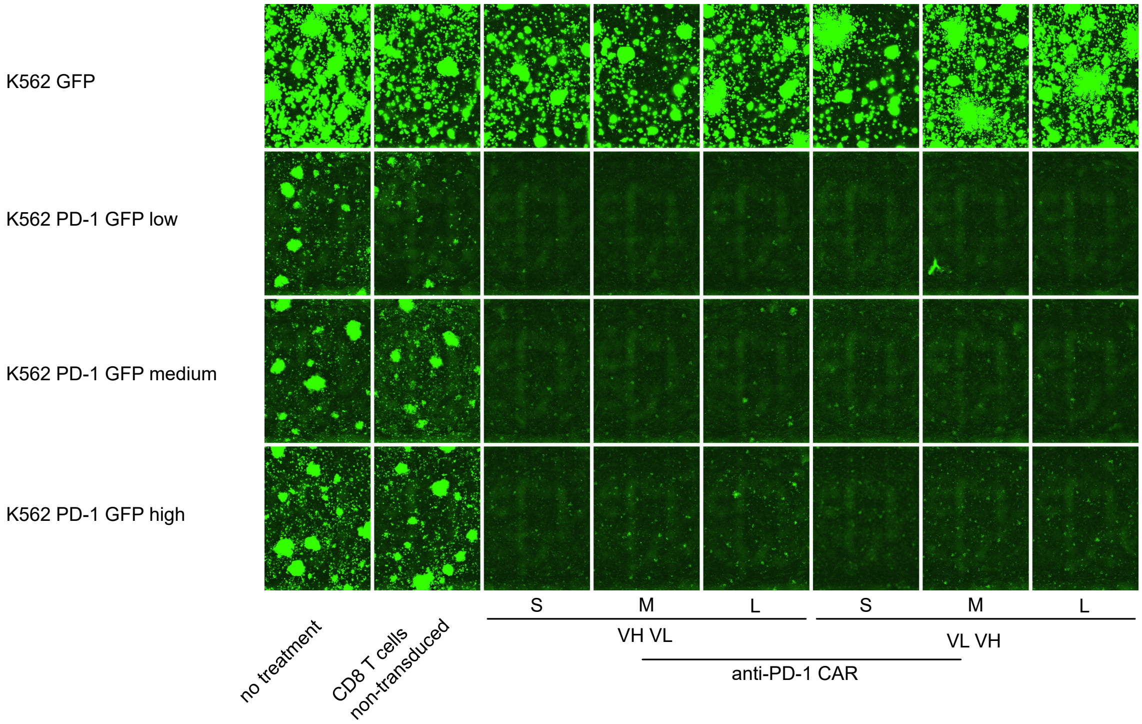

Supplemental Figure 5: Primary anti-PD-1 CAR T cells efficiently kill PD-1+ cells. Representative micrographs of the killing assay shown in Figure 1D (n=3) at 111 h. Primary anti-PD-1 CAR CD8+ T cells or non-transduced CD8+ T cells were co-cultured with K562 PD1 GFP with low, medium and high PD-1 expression or K562 GFP control target cells at a 1:1 ratio. Cytotoxicity was measured by reduction of GFP+ cells using the Incucyte live cell imaging system. Images were acquired with a 10× objective.

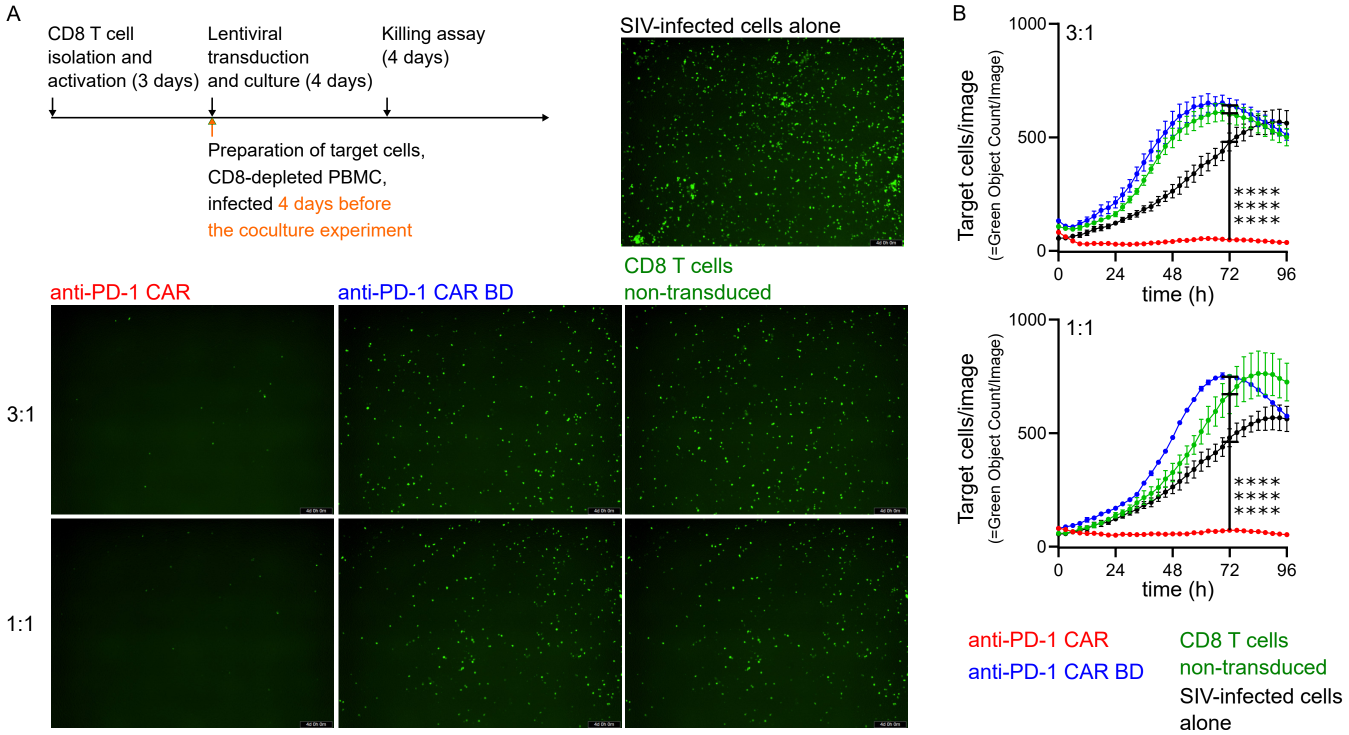

Supplemental Figure 6: Anti-PD-1 CAR T cells attenuate viral replication.
Schematic showing the timeline of preparation of effector cells (anti-PD-1 CAR, binding-deficient PD-1 CAR BD and non-transduced CD8+ T cells) and autologous CD4+ T cells as target cells infected with SIVmac239 NefIRESGFP for four days prior to experiment. Cytotoxicity was measured by reduction of GFP+ cells using the Incucyte live cell imaging system. Representative images of GFP+ SIV-infected CD4+ T cells at the end of the 96 h coculture with effector cells at the indicated E:T ratio (n=3). Images were acquired with a 10× objective (A). Quantification of GFP+ cells in the images acquired over 96 h in the killing assay described in (A). Average cytotoxicity ± SEM of one representative experiment is shown. Statistics were analyzed using a 2-way ANOVA with Tukey's multiple comparisons test at each time point. Results for 72 h are reported. **** = $p<0.0001$ (B).

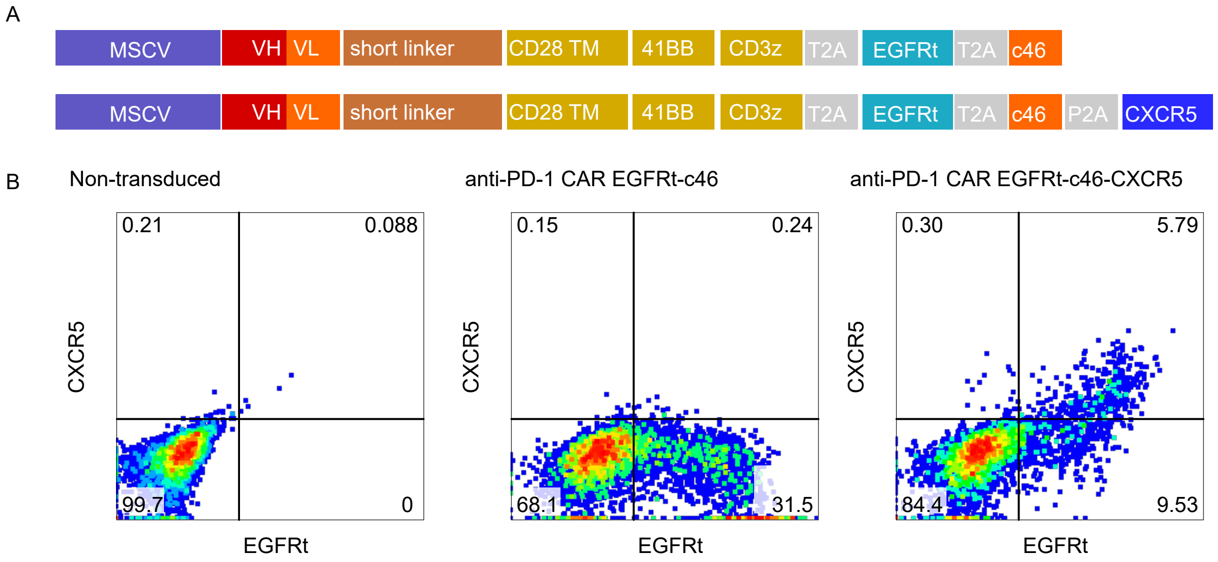

Supplemental Figure 7: Co-expression of CXCR5 and EGFRt in the anti-PD-1 CAR EGFRt c46 CXCR5 construct. Schematic of the anti-PD-1 CAR-EGFRt-c46 construct that co-expresses CXCR5 (A). Cell surface expression of EGFRt and CXCR5 on CD8+ T cells transduced with the indicated constructs (data from one representative experiment are shown, n=3) (B).

```
Macaca mulatta   PD-1  NP_001107830.1     MQIPQAPWPVVWAVLQLGWRPGWFLESPDRPWNPPTFSPALLLVTEGDNATFTCSFSNAS  60
Homo sapiens     PD-1  XP_016859782.1     MQIPQAPWPVVWAVLQLGWRPGWFLDSPDRPWNPPTFSPALLVVTEGDNATFTCSFSNTS  60
                                          *************************:****************:***************:*

Macaca mulatta   PD-1  NP_001107830.1     ESFVLNWYRMSPSNQTDKLAAFPEDRSQPGRDCRFRVTQLPNGRDFHMSVVRARRNDSGT  120
Homo sapiens     PD-1  XP_016859782.1     ESFVLNWYRMSPSNQTDKLAAFPEDRSQPGQDCRFRVTQLPNGRDFHMSVVRARRNDSGT  120
                                          ******************************:*****************************

Macaca mulatta   PD-1  NP_001107830.1     YLCGAISLAPKAQIKESLRAELRVTERRAEVPTAHPSPSPRPAGQFQALVVGVVGGLLGS  180
Homo sapiens     PD-1  XP_016859782.1     YLCGAISLAPKAQIKESLRAELRVTERRAEVPTAHPSPSPRPAGQFQTLVVGVVGGLLGS  180
                                          ***********************************************:************

Macaca mulatta   PD-1  NP_001107830.1     LVLLVWVLAVICSRAAQGTIEARRTGQPLKEDPSAVPVFSVDYGELDFQWREKTPEPPAP  240
Homo sapiens     PD-1  XP_016859782.1     LVLLVWVLAVICSRAARGTIGARRTGQPL-EDPSAVPVFSVDYGELDFQWREKTPEPPVP  239
                                          ****************:*** ******** ****************************.*

Macaca mulatta   PD-1  NP_001107830.1     CVPEQTEYATIVFPSGLGTSSPARRGSADGPRSPRPLRPEDGHCSWPL    288
Homo sapiens     PD-1  XP_016859782.1     CVPEQTEYATIVFPSGMGTSSPARRGSADGPRSAQPLRPEDGHCSWPL    287
                                          ****************:**************** :*************
```

Supplemental Figure 8: The amino acids crucial for the binding of pembrolizumab are well conserved between Rhesus macaque and human PD-1. Residues in red font are implicated to interact with anti-PD-1 antibody Pembrolizumab (20). Asterisks (*) indicate identical amino acids and period (.) and colon (:)indicate amino acids with similar properties. Grey background denotes the transmembrane domain. Alignment was prepared with the CLUSTAL O (1.2.4) multiple sequence alignment tool (21).

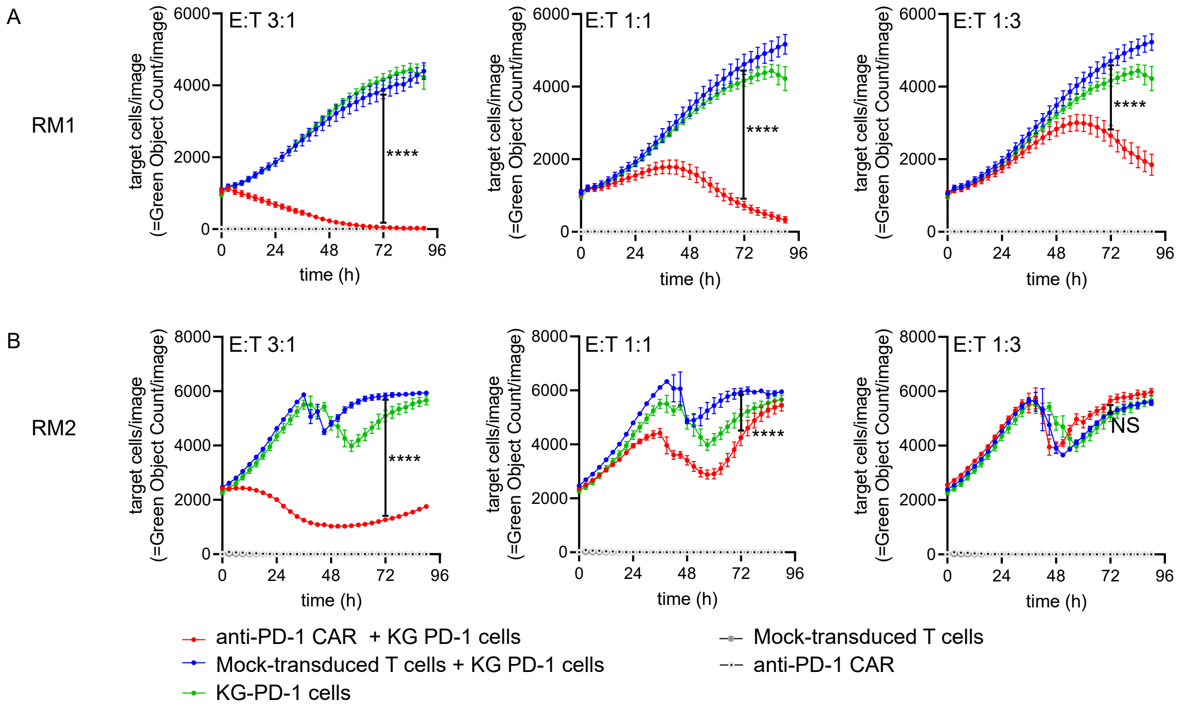

Supplemental Figure 9: Kill curves of ant-PD-1 CAR T cells infused in SIV-naive RMs. Infusate anti-PD-1 CAR T cells were cocultured with K562 GFP (KG) PD-1 cells at the indicated E:T ratio and GFP expression was followed in live cell microscopy killing assay. E:T ratio were calculated based %EGFRt of CD3+ cells in the infusion product. Average cytotoxicity ± SEM for individual infusion products for RM1 (A) and RM2 (B) are shown. Statistics were analyzed using a 2-way ANOVA with Tukey's multiple comparisons test at each time point. Results for 72 h are reported. **** = $p<0.0001$.

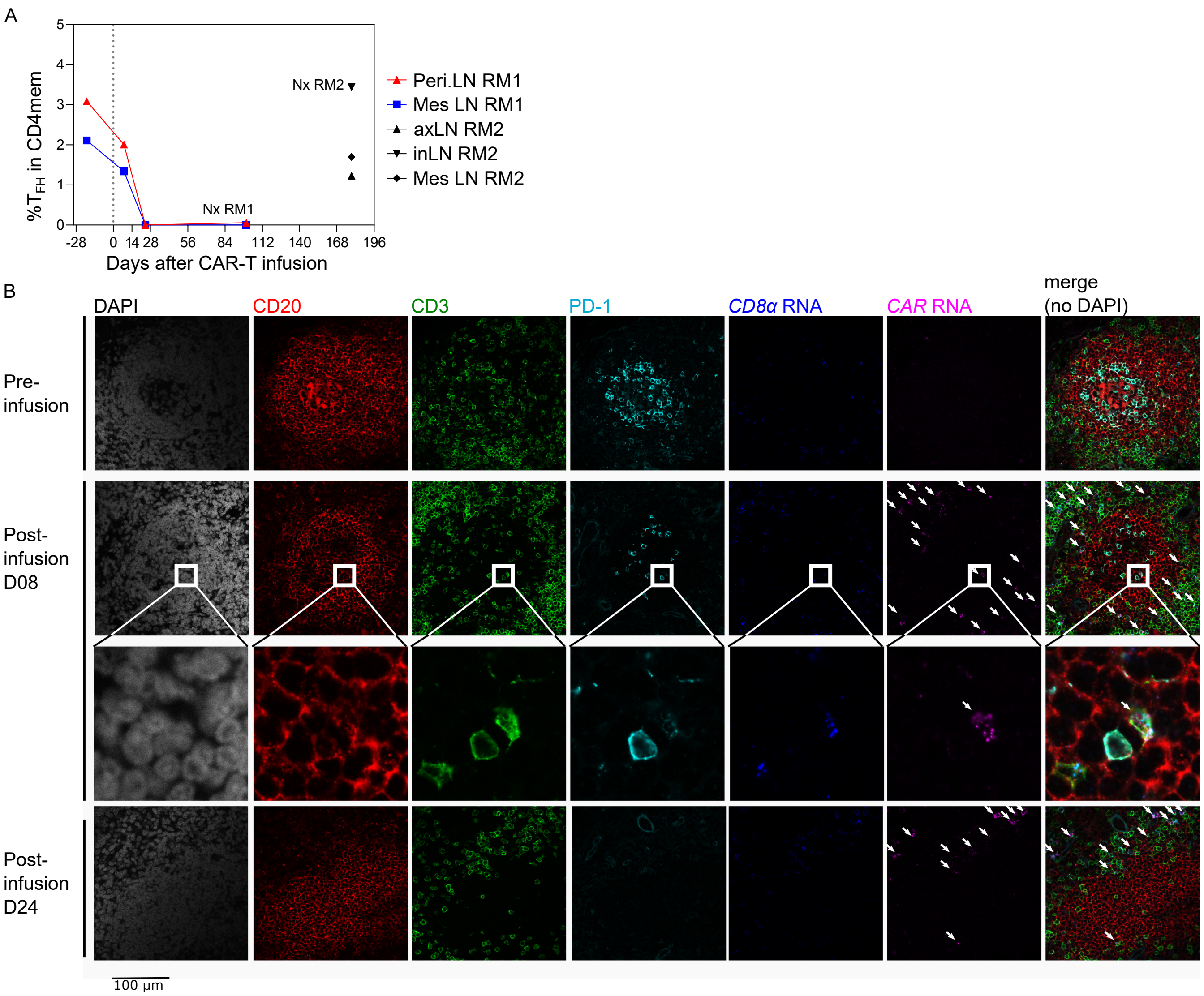

Supplemental Figure 10: Anti-PD-1 CAR T cell infiltrate lymph node germinal centers and mediate depletion of lymph node PD-1+ T_{FH} cells. Frequency of follicular helper T cells (T_{FH}) in CD4+ total memory T cells in peripheral and mesenteric LN in RM1 (CAR expanded) at various timepoints and RM2 (non-expanded, only necropsy (Nx) samples were available for analysis) (A). Multicolor immunofluorescence and RNA FISH staining for DAPI (grey), CD3 (green), CD20 (red), PD-1 (cyan), CD8α RNA (blue) and CAR RNA (magenta) (B). CD3+ PD-1+ cells in the follicles are considered TFH cells. These are the single-color images that were used to create the merged images in Figure 3H. Merged images are also shown on the right without the addition of the DAPI channel. White arrows indicate the location of anti-PD-1 CAR T cells. Images including zoomed-in sections were acquired with a 40× objective.

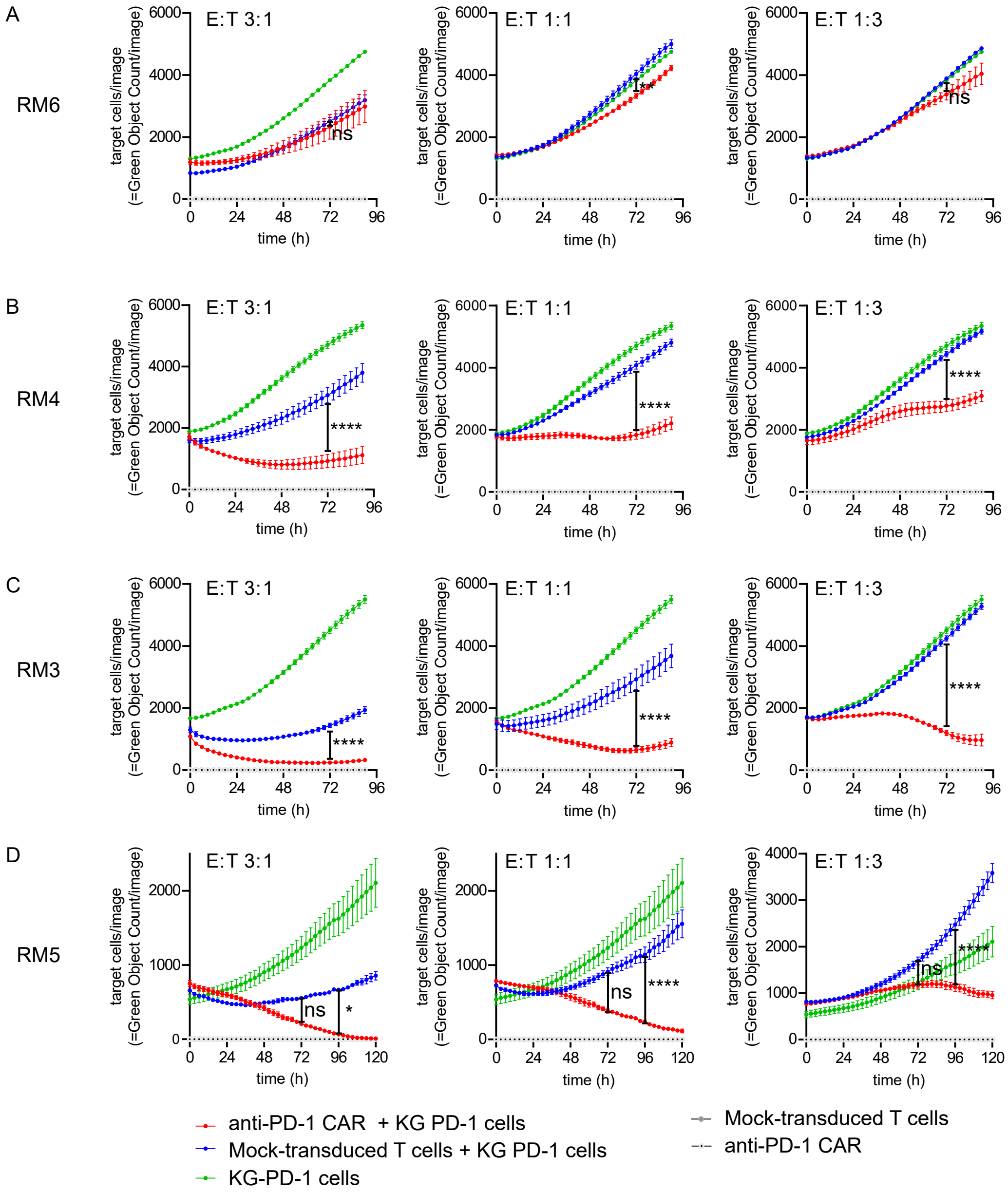

Supplemental Figure 11: Killing assays for the infusion products of the four SIVmac239-infected RM. Infusate anti-PD-1 CAR T cells were cocultured with K562 GFP (KG) PD-1 cells at the indicated E:T ratio and GFP expression was followed in a live cell microscopy killing assay. E:T ratio were calculated based on %EGFRt of CD3+ cells in the infusion product. Average cytotoxicity ± SEM for individual infusion products for RM6 (A), RM4 (B), RM3 (C) and RM5 (D) are shown. Statistics were analyzed using a 2-way ANOVA with Tukey's multiple comparisons test at each time point. Results for 72 h are reported. *=p<0.05, **= p<0.01, **** = p<0.0001.

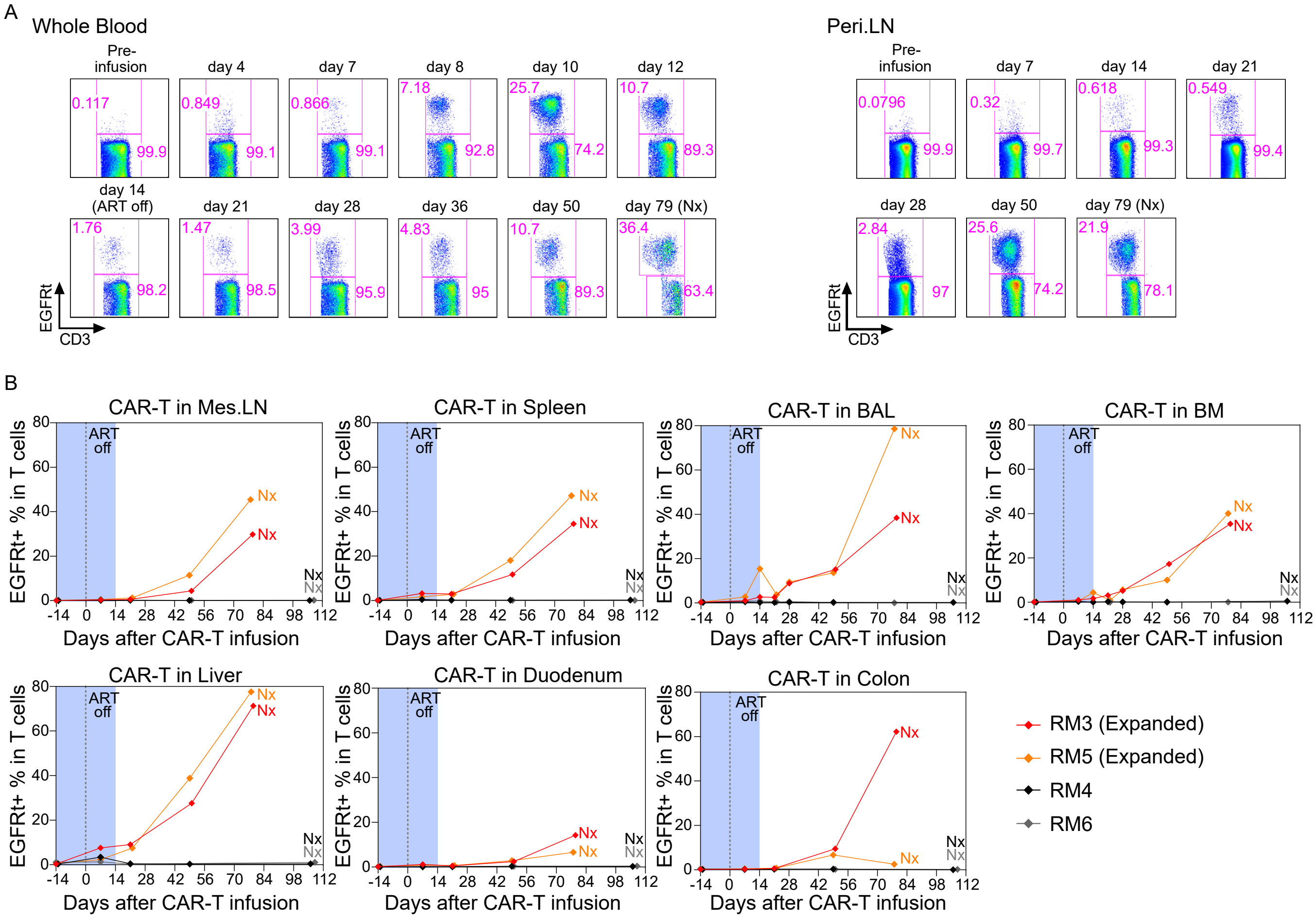

Supplemental Figure 12: Anti-PD-1 CAR T cell tissue traffic/expansion in various tissues. Flow pictures showing EGFRt+ T cells in whole blood and Peripheral lymph node (Peri.LN) in RM3 (A). Frequency of anti-PD-1 CAR of total T cells in the biopsy and necropsy tissues in the individual RM (B).

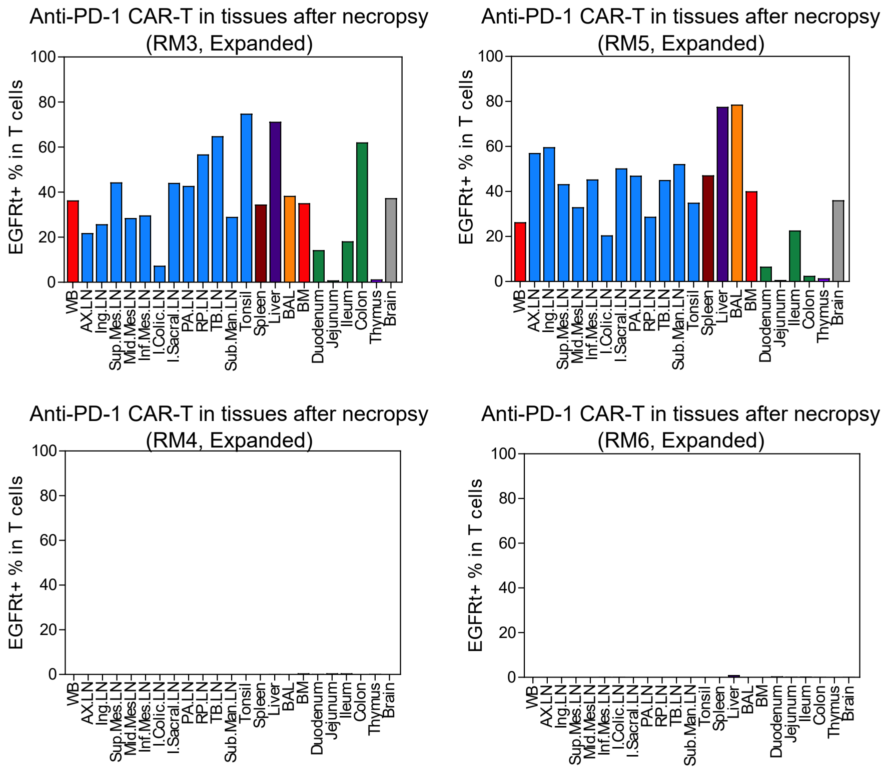

Supplemental Figure 13: Tissue distribution of anti-PD-1 CAR T cell at necropsy. Frequency of (EGFRt+) anti-PD-1 CAR T cells in the indicated tissues at necropsy in the indicated RM.

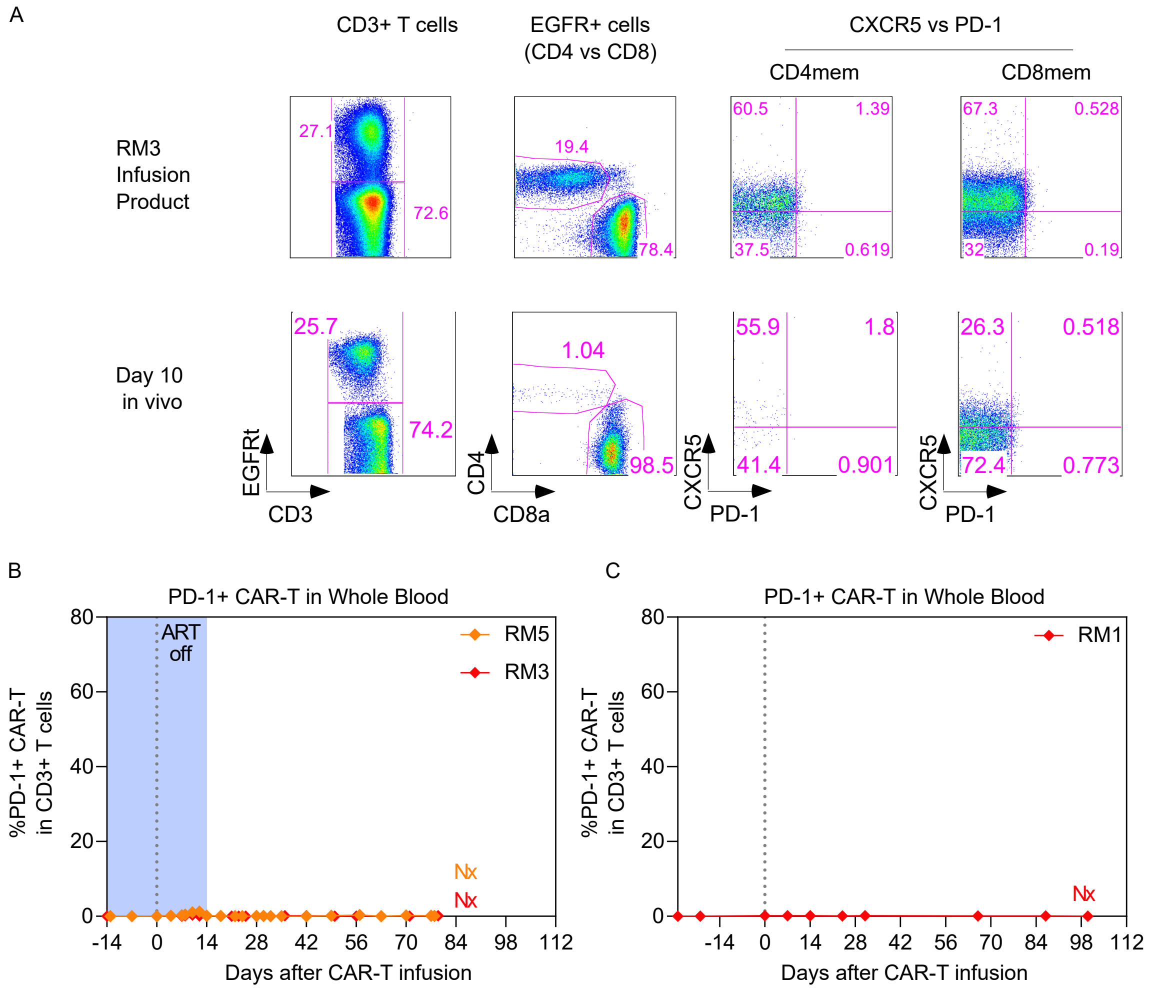

Supplemental Figure 14: Lack of PD-1 expression on anti-PD-1 CAR T cell infusion products and in vivo-expanded CAR T cells.

Representative flow cytometry data showing PD-1 expression on memory CD4+ and CD8+ T anti-PD-1 CAR T cells in the infusion product and in vivo expanded cells on day 10 (A). Frequency of PD-1+ EGFRt+ T cells in vivo in longitudinal PBMC of SIV-infected RM RM3 and RM5 and (B) SIV-naive RM RM1 (C).

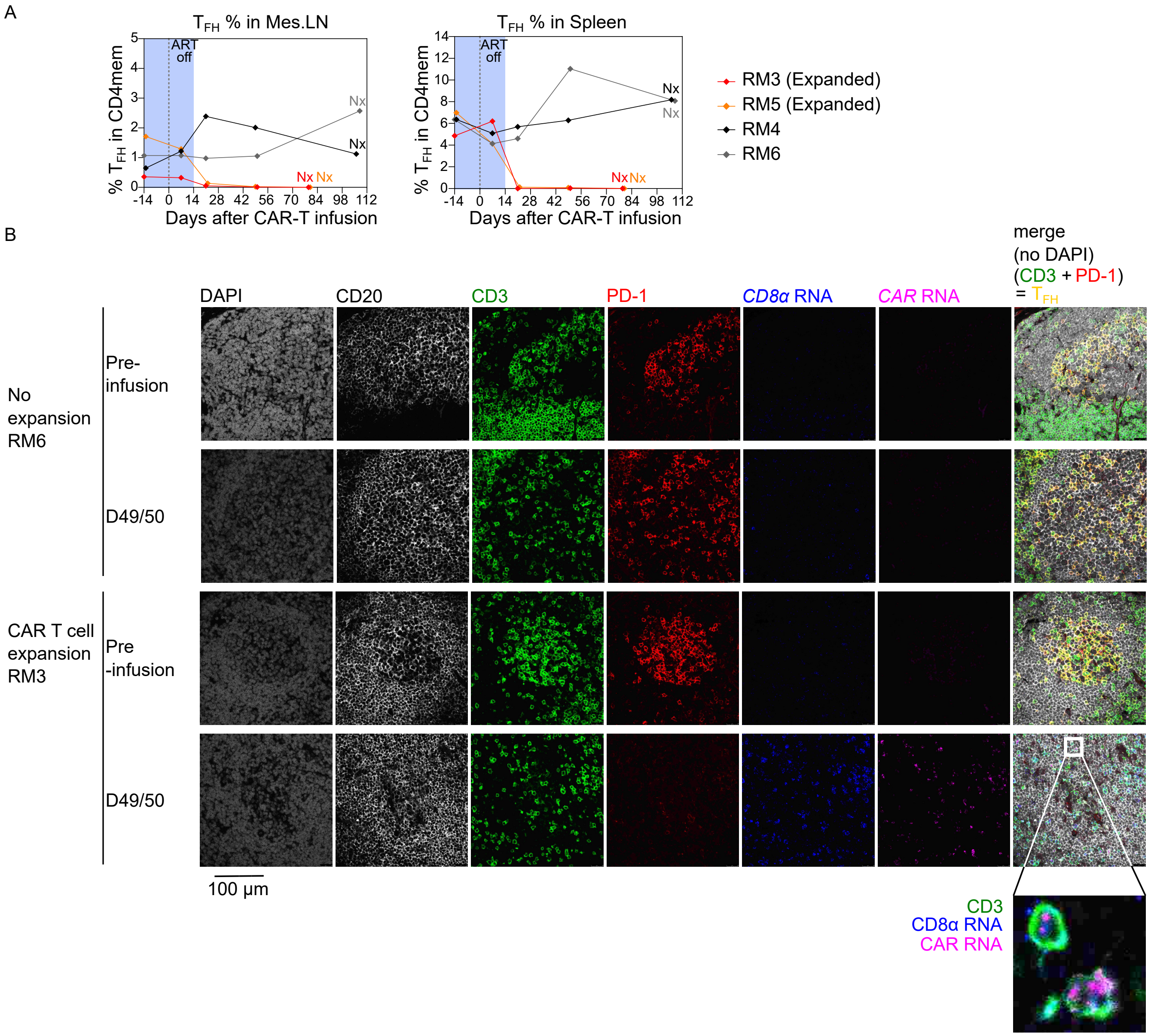

Supplemental Figure 15: CAR T cell-mediated depletion of lymph node PD-1+ T_{FH} cells. Frequency of T_{FH} (CXCR5+, PD-1hi) in CD4+ total memory T cells (A). Combined immunofluorescence and RNA FISH staining on lymph node tissue section from one animal without CAR T cell expansion and one animal with CAR T cell expansion (B). Single color staining is shown for DAPI (grey), CD3 (green), CD20 (grey), PD-1 (red), CD8α RNA (blue) and CAR RNA (magenta). Merged colors are shown without the addition of the DAPI channel. CD3+ PD-1+ cells in the follicles appear yellow and are defined as T_{FH} cells. Images including zoomed-in sections were acquired with a 40× objective.

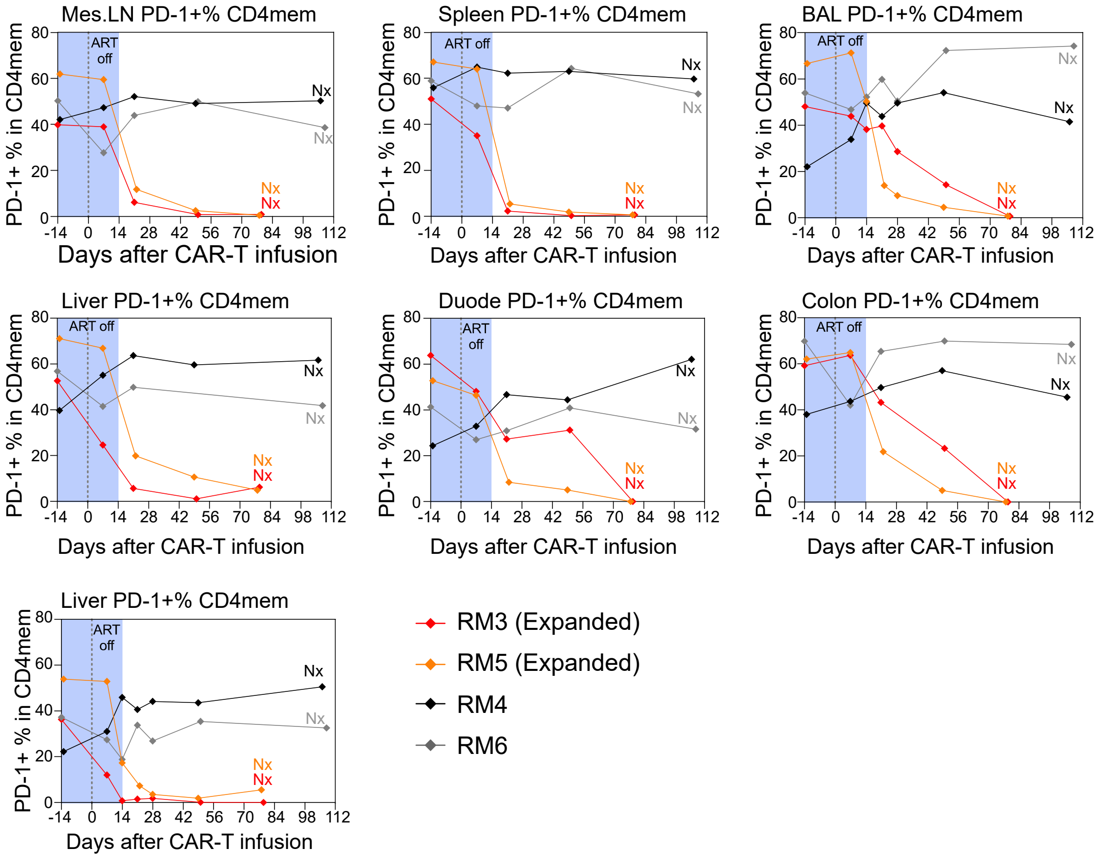

Supplemental Figure 16: Depletion of PD-1+ CD4+ T cells in various tissues. Frequency of PD-1+ cells in CD4+ total memory T cells in the indicated tissues.

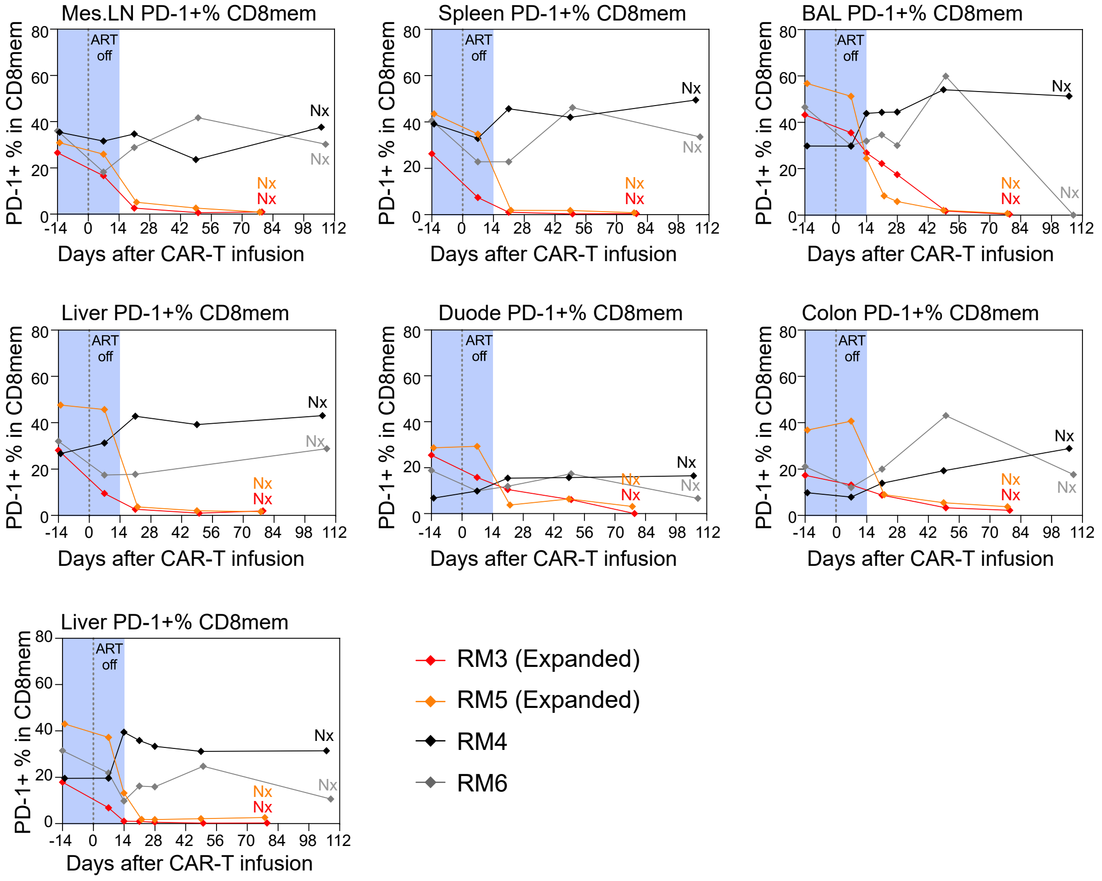

Supplemental Figure 17: Depletion of PD-1+ CD8+ T cells in various tissues. Frequency of PD-1+ cells in total CD8+ memory T cells in the indicated tissues.

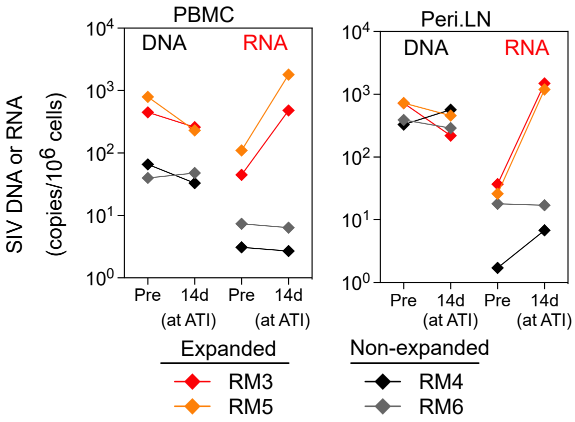

Supplemental Figure 18: Cell-associated RNA and DNA levels in PBMC and lymph node pre-infusion and 14 days post-CAR T cell infusion. PBMC and Peri.LN cell associated SIV DNA and RNA at the pre-anti-PD1-CAR-T cell infusion and at the time of ART cessation, 14 days post infusion.

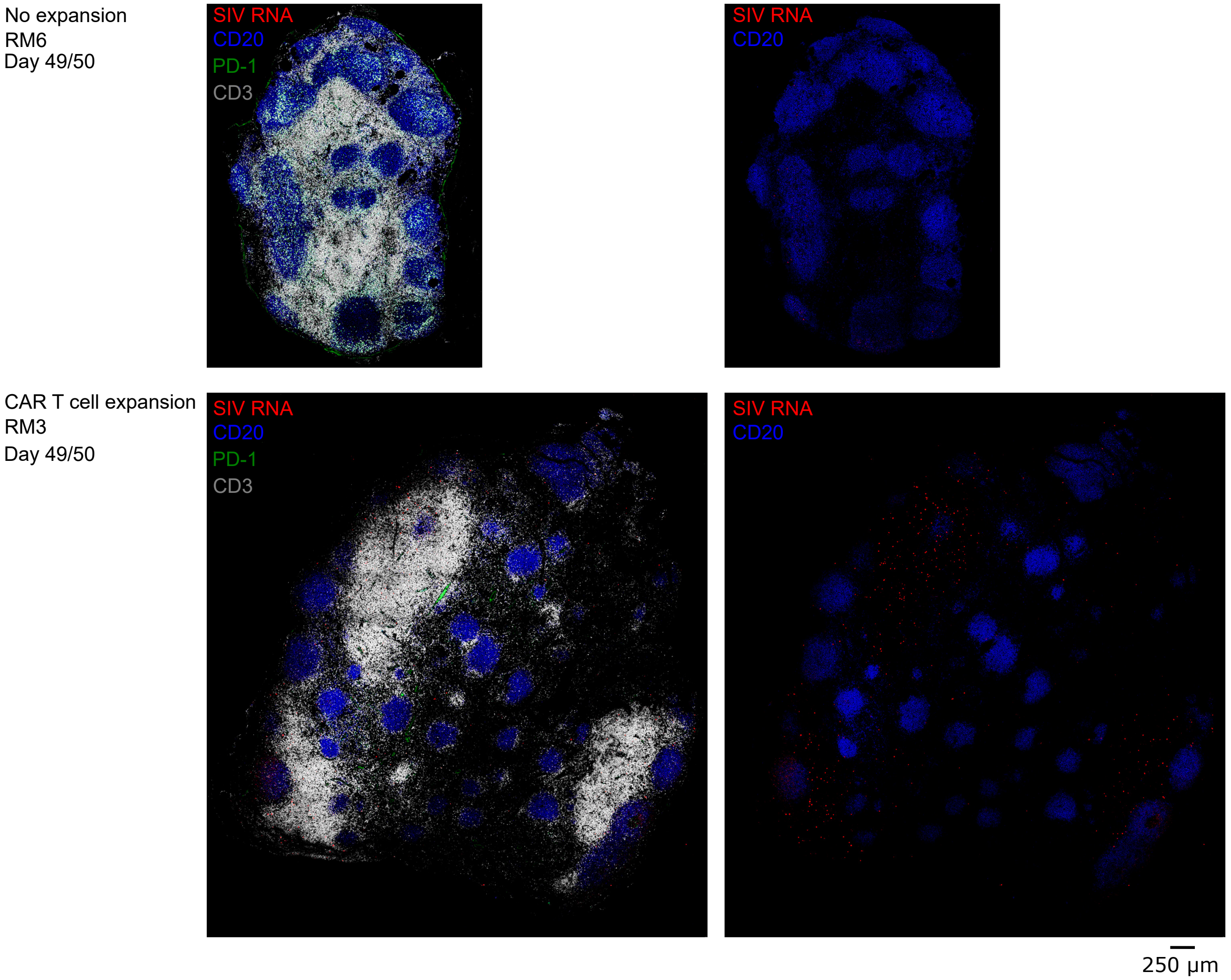

Supplemental Figure 19: Depletion of PD-1+ T cells in lymph nodes.
Combined immunofluorescence and RNA FISH staining on D49/50 lymph node tissue section from one animal without CAR T cell expansion and one animal with CAR T cell expansion for CD3 (grey), CD20 (blue), PD-1 (green), SIV RNA (magenta). The overlay CD20 and SIV RNA staining shows the enrichment of SIV infection in the extrafollicular T cell zone after successful anti-PD-1 CAR T cell expansion. Images were acquired with a 40× objective.

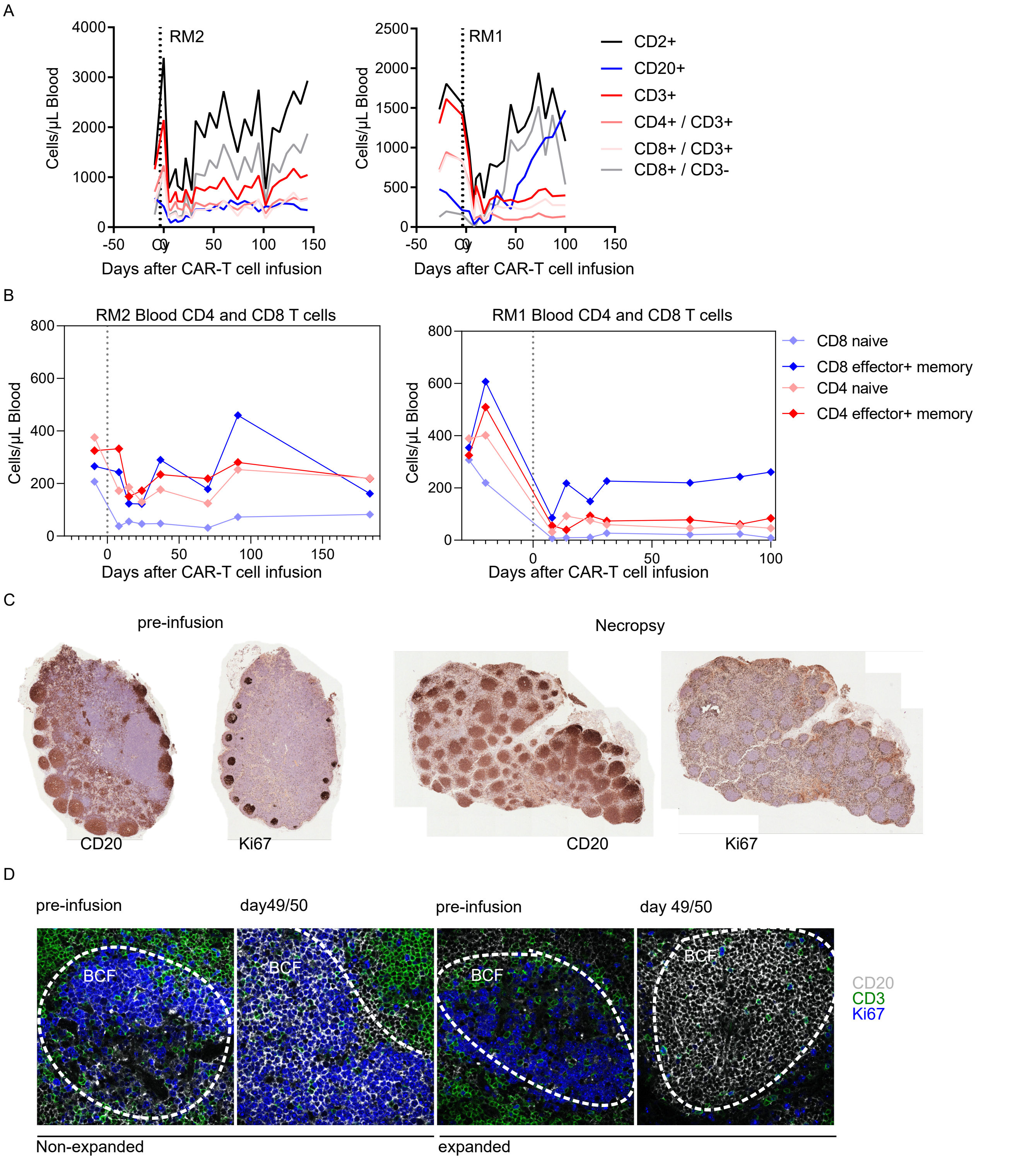

Supplemental Figure 20: Impact of anti-PD-1 CAR T cells on the T cell and B cell compartments. Absolute counts of CD2+, CD20+, CD3+, CD4+ and CD8+ cells in the SIV-naive RMs (A). Absolute count of CD4+ memory and CD8+ memory T cells in blood in the SIV-naive RMs (B). CD20 and Ki67 immunohistochemistry staining of pre-infusion and necropsy lymph node tissue section of RM1, an animal with anti-PD-1 CAR T cells expansion (C). CD3 (green), CD20 (grey) and

Ki67 (blue) immunofluorescence staining of pre-infusion and day 49/50 lymph node tissue sections of an RM without and an RM with CAR T cell-expansion. The white line demarcates the border between the T cell zone and the B cell follicle (BCF) (D). Images were acquired with a 40× objective.

Supplemental table 1: Characteristics of the six anti-PD-1 CAR T cell infusion products. Bold font indicates RM with CAR-T expansion.

Animal ID	Weight	CAR ID	SIV status	Profile of infused CAR-T cells							CAR-T expansion in vivo	
				Infusion day after lentiviral transduction	Total Infused T cells (10^6 cells/kg)	EGFRt+ CAR-T % in infused cells	Total infused CAR-T cells (10^6 cells)	Infused CAR-T cells (10^6 cells/kg)	CD4+% in EGFRt+ CAR-T	CD8+% in EGFRt+ CAR-T	Peak % EGFRt+ CAR-T in PBMC	Initial peak expansion (days Post infusion)
**RM1**	**9.4 kg**	**PD1**	**Negative**	**4 day**	**24.8**	**48.2%**	**112**	**12.0**	**55.8%**	**43.2%**	**58.40%**	**14**
**RM3**	**6.6 kg**	**PD1**	**Positive**	**4 day**	**77.0**	**27.0%**	**137**	**20.8**	**19.4%**	**78.4%**	**25.70%**	**10**
**RM5**	**12.2 kg**	**PD1-CXCR5**	**Positive**	**7 day**	**41.6**	**12.3%**	**82**	**6.7**	**33.0%**	**64.1%**	**13.90%**	**12**
RM2	8.7 kg	PD1	Negative	4 day	36.7	17.0%	52	6.0	42.4%	57.6%	6.49%	3
RM6	7.4 kg	PD1-CXCR5	Positive	4 day	66.7	18.0%	88	12.0	53.1%	41.4%	0.77%	7
RM4	7.6 kg	PD1	Positive	4 day	25.7	30.1%	59	7.7	17.7%	80.3%	0.68%	21

Supplemental table 2: Comparison of anti-PD-1 CAR T cell observations in SIV-naive and SIV-infected RMs.

	SIV-infected RM	healthy RM
CD4+ T cells	depletion of PD-1+ memory CD4+ T cells	depletion of PD-1+ memory CD4+ T cells
CD8+ T cells	depletion of PD-1+ memory CD8+ T cells	depletion of PD-1+ memory CD8+ T cells was less pronounced
lymphocytopenia		total CD4+ subset
GC B cells	loss of Ki67+ GC B cells	loss of Ki67+ GC B cells
germinal centers	underdeveloped	underdeveloped
Monocytes	Monocytosis	Monocytosis

Supplemental Table 3: Anti-PD-1 CAR T cell expansion and depletion of T_{FH} cells does not affect antibody recall responses.

Animal ID	Post-infusion timepoint (weeks)		**ID50[1] SVA-MLV**	**ID50[1] SIVmac251.6**
RM3	0		232	**11,969**
RM3	0.5		<200	**15,747**
RM3	1		<200	**11,299**
RM3	2		<200	**14,230**
RM3	5		<200	**124,828**
RM3	NX		<200	**92,906**
RM4	0		411	**3,530**
RM4	0.5		<200	**999**
RM4	1		<200	**4,269**
RM4	2		<200	**2,553**
RM4	5		<200	**43,802**
RM4	Nx		<200	**89,206**
RM6	0		395	**8,819**
RM6	0.5		<200	**8,534**
RM6	1		<200	**10,580**
RM6	2		<200	**20,858**
RM6	5		<200	**165,034**
RM6	Nx		<200	**204,072**
RM5	0		381	**20,557**
RM5	0.5		<200	**15,217**
RM5	1		<200	**10,037**
RM5	2		<200	**21,239**
RM5	5		<200	**367,931**
RM5	NX		<200	**52,799**

[1]Values are the purified IgG concentration or flowthrough dilutions at which relative luminescence units (RLUs) were reduced 50% compared to virus control wells (no test sample).

Values in **bold type** are considered positive for neutralizing antibody activity in the sample based on the criterion of >3X background signal against the negative control MLV-pseudotyped virus.

Supplemental table 4: Summary of adverse event and necropsy findings.

Animal ID	RM6	RM4	RM2	RM5	RM3	RM1
SIV status	Positive	Positive	Negative	Positive	Positive	Negative
CAR T cell expansion	No	No	No	**Yes**	**Yes**	**Yes**
Clinical observation	no significant findings	no significant findings	no significant findings	Hypoalbuminemia, anemia (HCT 27%, RBC 4.01)) w/ 1.8% reticulocytes, monocytosis, platelets were decreasing but still in the normal range.<br><br>diarrhea and low appetite<br>treated with enrofloxacin and cefazolin.<br><br>Weight hadn't decreased yet.	Slow healing wounds, monocytosis, hypoalbuminemia, treated with cefazolin	Monocytosis,<br><br>CD4+ lymphocytopenia<br>Slight anemia with decreased RBC<br><br>No weight loss
Autopsy report Final principal Diagnosis	1. Follicular hyperplasia and dysplasia, multifocal, mild to moderate, splenic white pulp<br>2. -Gastritis, diffuse, chronic, mild to moderate, plasmacytic and lymphocytic<br>3. Lymphofollicular hyperplasia, multifocal, minimal to mild, axillary lymph node and gut-associated lymphoid tissue of the stomach, duodenum, and jejunum	1. Follicular hyperplasia and dysplasia, multifocal to coalescing, moderate, spleen and axillary, inguinal, and sacral lymph nodes<br>2. Follicular hyperplasia, moderate, with pleomorphic leukocytes, tonsils<br>3. Follicular hyperplasia, mild to moderate, ileum and submandibular, ileocecal, and mesenteric lymph nodes	1. Mild to moderate, diffuse, eosinophilic, lymphoplasmacytic and histiocytic gastro-entero-colitis with enteric villar blunting and fusion, and with moderate to extensive, near-diffuse, large intestinal spirochetosis<br>2. Chronic-fibrous encapsulated, focal, deep dermal to subcutaneous abscess	1. Atypical leukocyte proliferation, moderate to marked, with multifocal tissue invasion, lymph nodes, spleen, bone marrow, lung, liver, pancreas, duodenum, jejunum, ileum, cecum, colon, kidneys, urinary bladder, scrotum<br>2. Lymphodepletion, multifocal, moderate to severe, lymph nodes and spleen<br>3. Enteritis, multifocal to transmural, chronic, severe, pleocellular<br>4. Colitis, multifocal, chronic, moderate, lymphoplasmacytic and neutrophilic, with crypt abscesses<br>5. Pleuropneumonia, diffuse, chronic, moderate, pleocellular with pleomorphic mononuclear leukocytes, type II pneumocyte hyperplasia, edema, and pleural fibrosis	1. Atypical leukocyte proliferation, chronic, moderate to marked, with lymphocyte depletion, thymus, spleen, and lymph nodes (retropharyngeal, submandibular, tracheobronchial, axillary, inguinal, superior mesenteric, middle mesenteric lymph nodes<br>2. Interstitial pneumonia and pleuritis, multifocal, chronic, moderate, severe, neutrophilic and histiocytic, necrotizing, with protein-laden alveolar macrophages and hemorrhage, focal intralesional foreign material, and scant cells with Cytomegaloviral intranuclear inclusion bodies<br>3. Hypercellularity, moderate, bone marrow	1. Mild to moderate, pulmonary, multifocal, arterial and arteriolar, nodular, subintimal to medial, connective tissue and smooth muscular proliferations: nodular pulmonary proliferative arteriopathy<br>2. Moderate, diffuse, eosinophilic, lymphoplasmacytic and histiocytic gastro-entero-colitis with enteric villar blunting and fusion, and with moderate to extensive, multifocal, large intestinal spirochetosis<br>3. Low to moderate follicular activity with generally sparsely populated lymphoid follicles: lymph nodes and spleen<br>4. Moderate, multifocal, adrenal, corticomedullary calcification
Histology comments	Staining for lymphocryptoviral RNA, carried out on select tissues,<br>revealed rare positive leukocytes in the spleen, axillary lymph node, and ileocecal junction. This indicates a minimally active opportunistic infection likely due to immunocompromise. Though lymphoid and follicular hyperplasia seen in the gastrointestinal tract and inguinal and mesenteric lymph nodes may be related to the experimental protocol, these were minimal to mild changes which can be seen as background lesions in rhesus macaques.	Staining for lymphocryptoviral RNA, carried out on select tissues, revealed rare positive leukocytes in the spleen, axillary lymph node, inguinal lymph<br>node, middle mesenteric lymph node, jejunum, and ileum. This indicates a minimal active opportunistic infection due to the experimental protocol. Follicular hyperplasia, present to some degree in most lymphoid tissues, was likely due to SIV infection. Florid follicular hyperplasia and dysplasia within the spleen, tonsil, and axillary and inguinal lymph nodes may similarly be attributed to retroviral inoculation, though an atypical proliferation cannot be ruled out. Gastritis is a common finding in rhesus macaques, and may have been a background lesion or secondary to potential immune changes associated with SIV infection.	The inflammatory component of diagnosis #1, which can cause diarrhea and potentially other sequelae thereof, represents typical changes in this species in this colony, and they have been previously discussed. Inflammatory changes present are consistent with food allergy/hypersensitivity/dietary intolerance/IBD. The bacterial organisms diagnosed should be considered commensals in my opinion.<br><br>As previously mentioned, the encapsulated abscess was an incidental lesion.	Atypical leukocyte proliferation was present in lymphoid and most major organs, largely associated with positive lymphocryptoviral (LCV) RNA staining were<br>assessed. The pleomorphic nature, high mitotic rate, presence in multiple tissues, and vascular invasion indicate a<br>neoplastic process of probable large cell lymphoma. LCV is a well-documented inciting cause of atypical lymphocytosis and non-Hodgkins-like lymphoma (NHL) in SIV-infected rhesus macaques. These lymphomas are typically, B cell-derived and hence CD20+. LCV NHLs, however, are primarily extra-nodal; an aspect in which this case Deviates. Though inciting agents were not able to be elucidated, inflammation in the lungs, duodenum, and colon, likely were related to opportunistic infections secondary to immunocompromise. Rare adenoviral infection within the jejunum and amoeba within the colon were not associated with significant inflammation, and so may be incidental or represent minimal opportunistic infections.	Moderate to marked atypical leukocyte proliferation was present in most lymph nodes are well as the spleen and thymus, which in areas disrupted the normal architecture. This was largely associated with positive lymphocryptoviral<br>(LCV) RNA staining where assessed. LCV staining was multifocally present within the tonsil and bone marrow. LCV is a well-documented inciting cause of atypical lymphocytosis and non-Hodgkins lymphoma (NHL) in SIV-infected rhesus macaques. As with another animal in this cohort, the pleomorphic leukocytic<br>proliferation was mostly present within lymph nodes, which would be abnormal for LCV NHLs that are generally extranodal. The histiocytic and neutrophilic pneumonia was caused by CMV, which is a common opportunistic infection in immunocompromised macaques. A small section of foreign material was present within one lung lesion, likely an incidental finding secondary to previous aspiration.	Histology Comments:<br>The nodular proliferative pulmonary arteriopathy was unexpected particularly as this lesion is typically associated with certain strains of lentivirus. In this case, consequences of the experimental manipulations is favored as the cause. The lesions were currently clinically silent, but could have progressed. The lymphoid follicular changes also are favored as being secondary to experimental manipulations.<br><br>The inflammatory component of diagnosis #2, which can cause diarrhea and potentially other sequelae thereof, represents typical changes in this species in this colony, and they have been previously discussed. Inflammatory changes present are consistent with food allergy/hypersensitivity/dietary intolerance/IBD. The bacterial organisms diagnosed should be considered commensals in my opinion.
